# The causal associations of circulating lipids with Barrett’s Esophagus and Esophageal Cancer: a bi-directional, two sample mendelian randomization analysis

**DOI:** 10.1186/s40246-024-00608-6

**Published:** 2024-04-16

**Authors:** Baofeng Li, Meng Li, Xiao Qi, Ti Tong, Guangxin zhang

**Affiliations:** Department of Thoracic Surgery, The Second Hospital of Jilin University, Changchun, Jilin 130021 China

**Keywords:** Barrett's Esophagus, Esophageal Cancer, Mendelian randomization, Circulating lipid, Triglycerides

## Abstract

**Objective:**

The causal associations of circulating lipids with Barrett’s Esophagus (BE) and Esophageal Cancer (EC) has been a topic of debate. This study sought to elucidate the causality between circulating lipids and the risk of BE and EC.

**Methods:**

We conducted two-sample Mendelian randomization (MR) analyses using single nucleotide polymorphisms (SNPs) of circulating lipids (*n* = 94,595 − 431,167 individuals), BE (218,792 individuals), and EC (190,190 individuals) obtained from the publicly available IEU OpenGWAS database. The robustness and reliability of the results were ensured by employing inverse-variance weighted (IVW), weighted median, MR-Egger, and MR-PRESSO methods. The presence of horizontal pleiotropy, heterogeneities, and stability of instrumental variables were assessed through MR-Egger intercept test, Cochran’s Q test, and leave-one-out sensitivity analysis. Additionally, bidirectional MR and multivariable MR (MVMR) were performed to explore reverse causality and adjust for known confounders, respectively.

**Results:**

None of the testing methods revealed statistically significant horizontal pleiotropy, directional pleiotropy, or heterogeneity. Univariate MR analyses using IVW indicated a robust causal relationship between increased triglycerides and BE (odds ratio [OR] = 1.79, p-value = 0.009), while no significant association with EC was observed. Inverse MR analysis indicated no evidence of reverse causality in the aforementioned outcomes. In MVMR analyses, elevated triglycerides (TRG) were significantly and positively associated with BE risk (OR = 1.79, p-value = 0.041).

**Conclusion:**

This MR study suggested that genetically increased triglycerides were closely related to an elevated risk of BE, potentially serving as a biomarker for the diagnosis of BE in the future.

**Supplementary Information:**

The online version contains supplementary material available at 10.1186/s40246-024-00608-6.

## Introduction

Esophageal cancer currently ranks as the sixth leading cause of cancer-related mortality characterized by a poor prognosis primarily attributed to early metastasis [1]. The 5-year survival rate for this condition remains below 40% [2]. Barrett’s esophagus (BE) is a condition characterized by the transformation of the normal squamous epithelium lining of the esophagus into specialized columnar cells. It represents the sole recognized precancerous lesion leading to the development of esophageal adenocarcinoma (EAC) [3]. Individuals diagnosed with BE face an elevated risk, ranging from 50 to 100 times higher, of developing malignant tumors compared to the general population [4]. Gastroesophageal reflux (GR) and body mass index (BMI) are known risk factors for esophageal cancer [5]. Chronic inflammation and tissue injury resulting from GR contribute to the development of BE [6]. Furthermore, extensive research has elucidated that the presence of metabolic syndrome significantly elevates the susceptibility to BE [7], subsequently increasing the risk of EAC [8].

Metabolic syndrome is characterized by the co-occurrence of various metabolic abnormalities, including obesity, insulin resistance, hypertension, and dyslipidemia [9]. There is a well-established correlation between metabolic syndrome and an elevated susceptibility to a wide range of malignancies, such as renal cell cancer, liver cancer, esophageal cancer, endometrial cancer, and pancreatic cancer, among others [10]. According to Aaron P et al., each 1 kg/m2 elevate in BMI is linked to a 10% and 20% elevated susceptibility of esophageal cancer (EC) and BE, respectively [11] Additionally, type 2 diabetes mellitus is also linked to EC [12] and BE [13] Dyslipidemia, a metabolic abnormality, is characterized by an imbalance in lipid profiles, including elevated levels of total cholesterol (TC), low-density lipoprotein (LDL) cholesterol, and triglycerides (TRG), along with decreased high-density lipoprotein (HDL) cholesterol. Previous studies have elucidated that elevated LDL cholesterol elevate the susceptibility of biliary tract cancer, while TRG decreases the risk [14]. LDL cholesterol has also been associated with breast cancer [15] However, existing research on the relationship between circulating lipids and the risk of BE and EC has yielded conflicting results [16, 17] Observational studies are prone to inherent limitations such as residual confounding and reverse causality [14]. Large-scale randomized clinical trials assessing the influence of circulating lipids on the risk of BE and EC are lacking. Within this context, Mendelian randomization (MR) techniques emerge as a valuable and robust alternative. MR analysis explores causality between exposure and outcome by leveraging genetic variants that are associated with the exposure variable under investigation [18]. As the random assortment of allelic genes during meiosis remains unaffected by disease processes, MR analysis helps mitigate the biases encountered in observational research and external interference [19]. Therefore, the aim of this study is to employ two-sample MR (univariate MR) and multivariable MR (MVMR) to investigate the causal relationship between genetically predicted levels of circulating lipids, including LDL, HDL, TG, and total cholesterol, and the risk of developing BE and EC.

## Methods and materials

### Study design and data source

The study employed a two-sample MR method to explore the causal effects of circulating lipids on BE and EC outcomes. The study design adhered to the three assumptions of MR design: (1) the instrumental variables chosen were strongly associated with circulating lipids, (2) these instrumental variables were not related to confounding factor, and (3) these instrumental variables were associated with BE and EC only when the effects were mediated by the exposure. The summary data of single-nucleotide polymorphisms (SNPs) was obtained from the IEU Open GWAS database (https://gwas.mrcieu.ac.uk, accessed on 20 January 2023). This database comprises 245,394,206,850 genetic associations from 42,335 GWAS summary datasets, which were available for querying or download.

Table 1 provides detailed information on the SNPs related to the plasma lipids, BE, EC, and confounding factors (BMI and GR). The study utilized summary statistics obtained from various databases for different variables. The summary statistics for HDL cholesterol were derived from the IEU GWAS database by Willer CJ, with a sample size of 94,595 (ebi-a-GCST002223) [20]. The summary statistics for LDL cholesterol were sourced from the IEU GWAS database by Klimentidis YC, involving a sample size of 431,167 (ebi-a-GCST90002412) [21]. The statistical data summarizing the association of triglyceride levels was acquired from the IEU GWAS database, also by Willer CJ, with a sample size of 94,595 (ebi-a-GCST002216) [20]. All lipid GWAS data were adjusted for gender age, etc., as detailed in the original literature.

**Table 1:** Characteristics of instrumental variable of exposure, outcome and confounders used for MR analysis

Type of variables	GWAS ID	Year	Trait	PMID	Author	Consortium	Population	Sample size	Number of SNPs
Exposure	ebi-a-GCST002223	2013	HDL cholesterol	24,097,068	Willer CJ	NA	European	94,595	2,418,527
	ebi-a-GCST90002412	2020	LDL cholesterol	32,493,714	Klimentidis YC	NA	European	431,167	16,293,344
	met-d-Total_C	2022	Total cholesterol	NA	Borges CM	NA	European	115,078	12,321,875
	ebi-a-GCST002216	2013	Triglycerides	24,097,068	Willer CJ	NA	European	94,595	2,410,057
Outcomes	finn-b-C3_OESOPHAGUS	2021	Esophageal cancer	NA	NA	NA	European	218,792	16,380,466
	finn-b-K11_BARRET	2021	Barret Esophagus	NA	NA	NA	European	190,190	16,380,373
Confounders	ebi-a-GCST90000514	2021	Gastroesophageal reflux	34,187,846	Ong JS	NA	European	602,604	2,320,781
	ukb-b-2303	2018	Body mass index	NA	Ben Elsworth	MRC-IEU	European	454,884	9,851,867

MR, Mendelian randomization; GWAS, genome-wide association studies; SNPs, single-nucleotide polymorphisms; NA, not available

The summary statistics for total cholesterol originated from the GWAS database by Borges CM, and consisted of 12,321,875 SNPs (met-d-Total_C). For the dataset of BE, the summary statistics with a sample size of 190,190 were obtained from FinnGen biobank (finn-b-K11_BARRET). For the dataset of EC, the summary statistics with a sample size of 218,792 were acquired from FinnGen biobank (finn-b-C3_OESOPHAGUS). The related SNPs of confounders (GR [22] and BMI) were acquired from the IEU OpenGWAS database.

### Univariable MR

GR [23] and BMI [12] were major risk factors of BE and EC. The SNPs related to GR and BMI were acquired from the IEU Open GWAS database. The GR dataset consisted of 129,080 individuals with gastroesophageal reflux and 473,524 control individuals. The BMI dataset included 454,884 participants. To mitigate the potential influence of these confounding factors, the MR analysis was conducted following the exclusion of IVs associated with either of these conditions. A subsequent reverse MR analysis was performed to address potential biases due to reverse causality. For the selection of instrumental variables, a p-value threshold of < 1 × 10^−5^ was employed, considering the absence of SNPs meeting the conventional GWAS criteria. Additionally, data clumping with parameters of R^2^ = 0.01 and kb = 5000 was utilized in the IV selection process.

### Multivariable MR

**Fig. 1:**
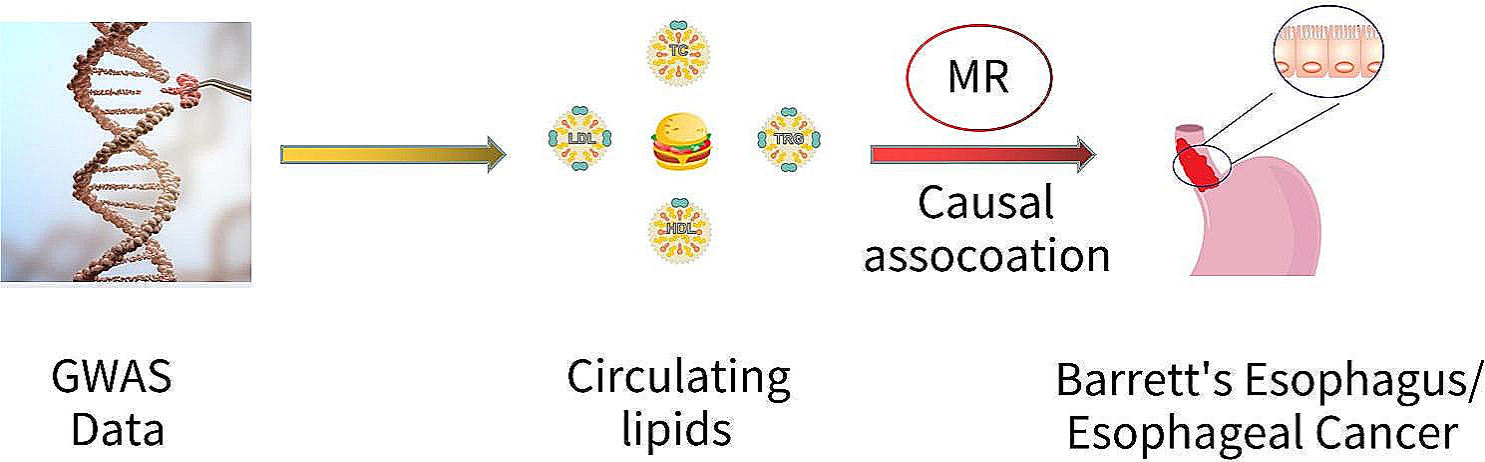
Flow chart of MR analysis in this study. MR, Mendelian randomization; BE, Barrett’s Esophagus; EC, esophageal cancer, GWAS, Genome-wide association studies

A comprehensive MVMR analysis was employed to address the intercorrelations among four lipid traits. By utilizing MVMR, we were able to estimate the causal effects of multiple exposures on the outcome of interest, while also facilitating the examination of the direct impact of individual exposures within the model [24]. The graphical representation of the study flow was illustrated in Fig. 1.

### Statistics

SNPs of exposure that reached genome-wide significance (p-value < 5 × 10 − 8) and showed no linkage disequilibrium (LD) (R^2^ < 0.001 and clump distance > 10,000 kb) were selected as instrumental variables. We harmonized the datasets for circulating lipids and BE/EC, while excluding SNPs with palindromic characteristics. The primary analysis was conducted utilizing the inverse variance weighted (IVW) method, which served as the main statistical approach. Additionally, supplementary analyses were performed employing the weighted median (WM) and MR-Egger methods, both implemented within the TwoSampleMR framework [25]. The MR-Egger regression was used to evaluate pleiotropy by examining the intercept p-value [26]. To identify and remove horizontal pleiotropic outliers, we employed the MR-PRESSO method [27]. The heterogeneity of the MR analysis was assessed using Cochran’s Q test [28]. To estimate the robust association of circulating lipids with BE and EC, we conducted a leave-one-out analysis. Associations with a Bonferroni-corrected p-value of IVW-based p-value < 0.0125 (0.05/4 risk factors) were considered meaningful. Associations with p-values ranging from 0.0125 to 0.05 were considered to indicate potential suggestive associations, implying a noteworthy but not definitive relationship between the variables under investigation. In order to avoid weak instrument bias (F < 10) in the two-sample model, we estimated the exposure strength of the instrumental variable using the approximation of the F statistic. The calculation of the F value and R^2^ adhered to the formula utilized in previous studies [29, 30]. We used the online tool mRnd to estimate the statistical power of the causal effect between exposure and outcomes (https://shiny.cnsgenomics.com/mRnd/) [31]. The statistical analyses described above were performed using R 4.2.2 software with TwoSampleMR version 0.5.6 and MRPRESSO version 1.0 package.

## Results

### Instrument variables

A total of 51, 74, 284, and 47 SNPs were identified as potential instrumental variables for TC, HDL, LDL, and TRG, respectively. We ensured that the statistical power for each MR analysis, as calculated by mRnd, was sufficient (100%). For detailed information on the instrumental variables, please refer to the supplementary materials (Table S1–S4).

### Univariable MR

**Fig. 2:**
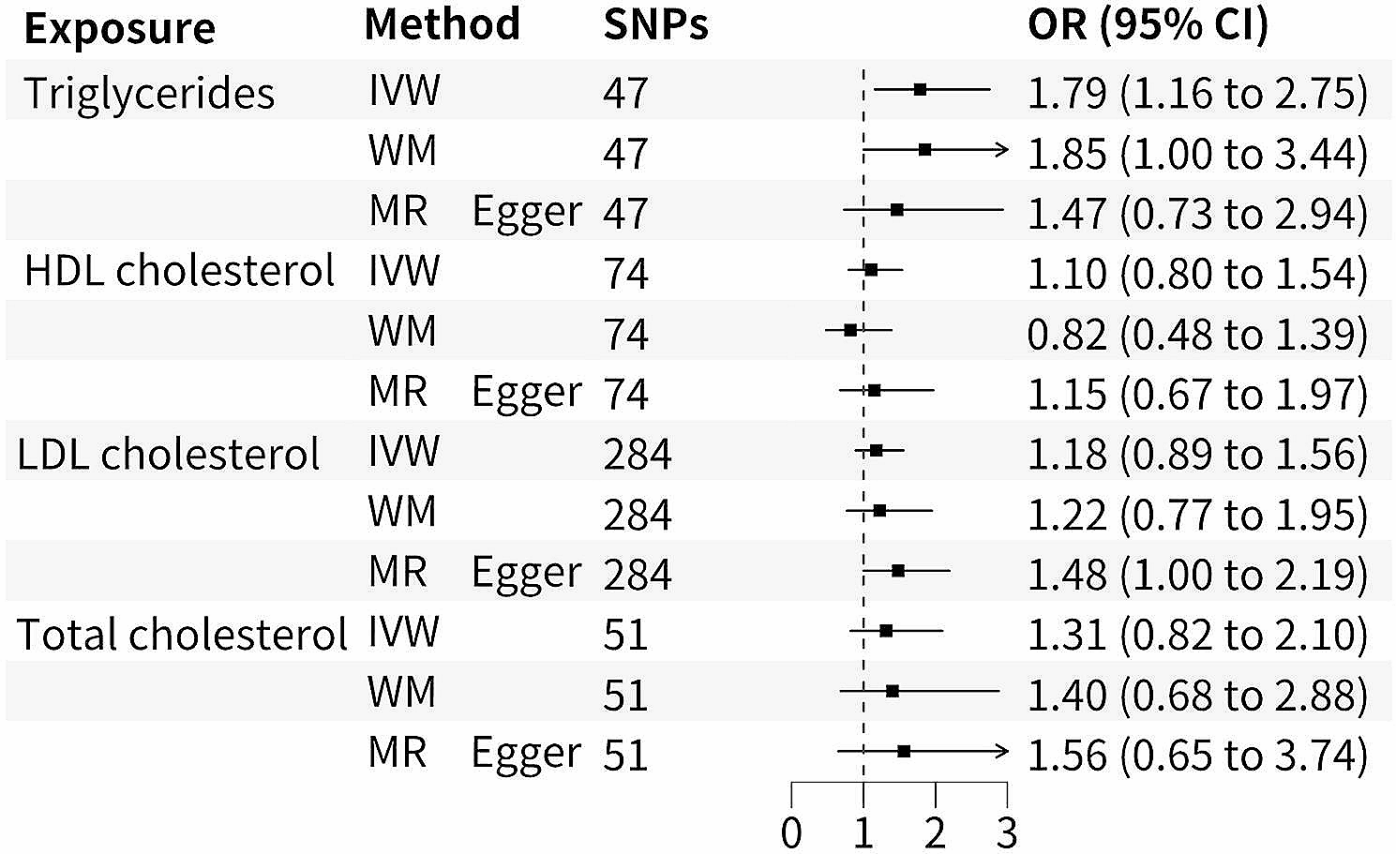
Forest plot of causal associations between circulating lipids and Barrett’s Esophagus outcomes. LDL, low density lipoprotein; HDL, high-density lipoprotein

**Fig. 3:**
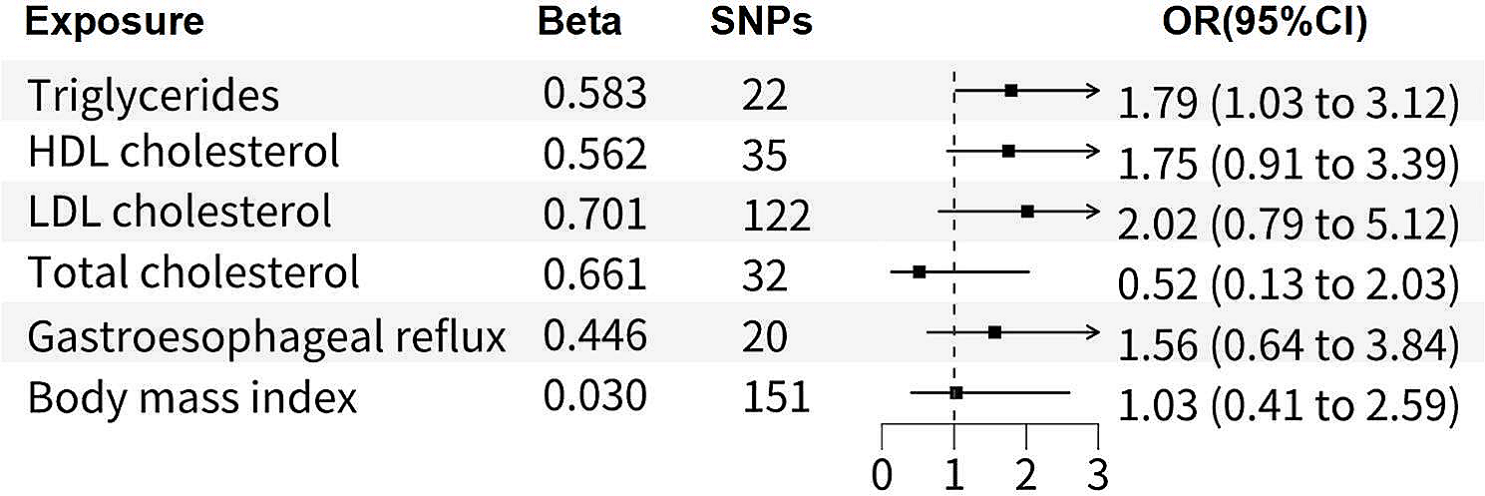
Forest plot of causal associations of MVMR analysis between circulating lipids and Barrett’s Esophagus outcomes

As shown in Fig. Fig3, the analysis revealed a significant correlation between triglyceride levels and an elevated susceptibility to BE, indicating that higher triglyceride levels were associated with an increased risk of developing this condition (p-value = 0.009, odds ratio [OR] = 1.79, 95% confidence interval [CI] = 1.16–2.75). However, no significant associations were observed between total cholesterol (p-value = 0.256, OR = 1.31, 95%CI = 0.82–2.10), HDL cholesterol (p-value = 0.550, OR = 1.10, 95%CI = 0.80–1.54), or LDL cholesterol (p-value = 0.250, OR = 1.18, 95%CI = 0.89–1.56) and the risk of BE using the IVW method (Fig. 2, Table S5). In addition, the analysis did not reveal any significant associations between genetically predicted TC (p-value = 0.819, OR = 0.92, 95%CI = 0.47–1.83), HDL (p-value = 0.341, OR = 1.27, 95%CI = 0.78–2.07), LDL (p-value = 0.922, OR = 0.98, 95%CI = 0.66–1.45), and TRG (p-value = 0.950, OR = 1.02, 95%CI = 0.55–1.91) and the risk of EC. These findings suggested that genetically determined cholesterol and TRG levels did not significantly contribute to the development of EC. (Table S6). Tests such as Cochran’s Q test (p-value > 0.05), MR Egger intercept test (p-value > 0.05), and leave-one-out analysis (p-value > 0.05) indicated no evidence of heterogeneity, directional pleiotropy, or robustness issues in both forward and reverse MR analyses (Table 2, Table S7, Figures S1–S10). The inverse MR analysis demonstrated no causality between circulating lipids and the risk of BE and EC (Supplementary Table S8–S9).

**Table 2:** The outcomes of sensitivity MR analyses of circulating lipids on Barrett’s Esophagus

Exposure	MR-PRESSO	IVW estimates		MR-Egger pleiotropy test	
	Global p-value	Cochran’s Q	P-value	MR-egger intercept	p-Value
Triglycerides	0.98	28.97	0.98	0.01	0.48
HDL cholesterol	0.40	74.47	0.43	-0.003	0.85
LDL cholesterol	0.28	297.29	0.27	-0.01	0.11
Total cholesterol	0.21	57.71	0.21	-0.01	0.65

MR, Mendelian randomization

### Multivariable MR

To address the potential issue of shared genetic instruments among circulating lipids, we conducted a MVMR analysis to elucidate the genetically predicted association between circulating lipids and BE. The comprehensive MVMR analysis, encompassing BMI and GR, shown a significant causality between elevated TRG levels and an increased likelihood of BE (p-value = 0.041, OR = 1.79, 95%CI = 1.03–3.122) (Fig. 3). However, no significant associations were observed between TC, LDL, HDL, and the risk of BE. We performed additional MVMR analyses between TRG and other lipids, which yielded similar conclusions (Table S11). Furthermore, the results indicated that plasma lipids were not linked to the risk of EC (Table S12, S13).

## Discussion

To our knowledge, the present study made the first attempt to prove the causal relationship between circulating lipids and the risk of BE and EC. Our study clarified the convincing influence of circulating lipids on BE, which indicated that increased triglycerides elevated the risk of BE. Since no pleiotropy and heterogeneity were detected by several methods, the results were considered robust.

Triglycerides, which form a chylomicron complex comprising lipids derived from food and absorbed by the intestinal lymphatics, played a significant role in previous study [32]. Moreover, the conversion products of triglycerides, such as lysophosphatidic acids, have been closely associated with tumor occurrence and development [33]. Specifically, lysophosphatidic acid has been implicated in mediating the progression of esophageal squamous cell cancer through the PI3K/Akt pathway [34]. Additionally, Akihiro et al. reported a significant causal link between hypertriglyceridemia and nodal metastasis of superficial esophageal carcinoma [16]. However, Xie et al. found no relationship between triglycerides and the risk of BE and EAC [17] It was important to note that inherent limitations in observational studies, such as measurement errors in lipid assessment, differences in participant demographics, and external confounding factors, can contribute to discrepant results [14] In contrast, MR analysis, employed in our study, was less susceptible to reverse causal effects and confounding factors [35]. To uphold the credibility and robustness of our findings, this study excluded SNPs related to BMI and GR, which were major confounding factors, and performed MVMR to address potential interactions among circulating lipids.

In this study, the GWAS statistics concerning lipid traits, BE and EC were retrieved from EBI GWAS database. Using genetic variants as instruments to conduct MR analysis, this study assessed the relationship between four lipids and the risk of BE and EC. Contrary to previous study that the levels of triglycerides were not associated with the risk of BE and EAC [17], our results suggested high levels of triglycerides had significantly relationship with BE, whereas no significant association was detected between LDL, HDL, and total cholesterol level and BE risk. Reverse MR analyses illustrated that there was no reverse causality between the levels of triglycerides and BE risks. Furthermore, the MVMR results, which adjusted for BMI and other lipids, confirmed a significant causality between genetically elevated triglyceride levels and an augmented risk of BE, which was not previously reported in the literature.

These outcomes were verified through various sensitivity analyses, heterogeneity analyses, and pleiotropy analyses, while excluding weak instrumental variables, thus ensuring the robustness of the findings. Additionally, we constructed an MVMR framework that adjusted for BMI, GR and other lipids to further confirm the significant relationship between triglyceride levels and BE risk. However, there were three limitations to consider in this study. Firstly, the genetic data used in this study was derived from European populations, and its applicability to other populations might be limited [14]. Secondly, besides BMI and gastroesophageal reflux, there might be other potential confounders that could influence the causal associations identified in this study. The consolidating factors primarily arise from the pleiotropy of IVs. In instances where no statistical differences were observed in the pleiotropy analysis, we concluded that the IVs did not exhibit pleiotropy, indicating no associations with other phenotypes. Fortunately, the pleiotropy analysis conducted using the MR method did not yield statistically significant results, ensuring the accuracy of our findings. Lastly, due to limitations in the available database, we did not analyze the relationship between triglyceride levels and EAC, which was closely related to BE.

## Conclusion

In this study, we utilized large-scale GWAS data to perform MR analysis, investigating the relationship between serum lipids and the risk of developing BE and EC. Our study provided compelling evidence that genetically determined elevated triglyceride levels were significantly associated with an increased risk of Barrett’s esophagus, as demonstrated by both UVMR and MVMR analyses. These findings hold great significance for the prevention of BE in future clinical practice, as they offer the potential to serve as pre-diagnostic markers. Furthermore, our research highlights the importance of regular monitoring of triglyceride levels for patients with esophageal diseases, such as gastroesophageal reflux, to mitigate disease progression resulting from prolonged high triglyceride levels.

### Electronic supplementary material

Below is the link to the electronic supplementary material.

Supplementary Material 1

## Data Availability

GWAS summary datasets of Barrett’s esophagus, esophageal cancer and circulating lipids were available on the IEU Open GWAS database (https://gwas.mrcieu.ac.uk).

## References

[1] Bray F, Ferlay J, Soerjomataram I (2018). Global cancer statistics 2018: GLOBOCAN estimates of incidence and mortality worldwide for 36 cancers in 185 countries. CA Cancer J Clin.

[2] Allemani C, Matsuda T, Di Carlo V (2018). Global surveillance of trends in cancer survival 2000-14 (CONCORD-3): analysis of individual records for 37 513 025 patients diagnosed with one of 18 cancers from 322 population-based registries in 71 countries. Lancet.

[3] Inadomi J, Alastal H, Bonavina L (2018). Recent advances in Barrett’s esophagus. Ann N Y Acad Sci.

[4] Zhang Y (2013). Epidemiology of esophageal cancer. World J Gastroenterol.

[5] Huang FL, Yu SJ (2018). Esophageal cancer: risk factors, genetic association, and treatment. Asian J Surg.

[6] Abrams JA, Fields S, Lightdale CJ (2008). Racial and ethnic disparities in the prevalence of Barrett’s esophagus among patients who undergo upper endoscopy. Clin Gastroenterol Hepatol.

[7] Drahos J, Ricker W, Parsons R (2015). Metabolic syndrome increases risk of Barrett esophagus in the absence of gastroesophageal reflux: an analysis of SEER-Medicare Data. J Clin Gastroenterol.

[8] Drahos J, Ricker W, Pfeiffer RM (2017). Metabolic syndrome and risk of esophageal adenocarcinoma in elderly patients in the United States: an analysis of SEER-Medicare data. Cancer.

[9] Engels EA, Pfeiffer RM, Ricker W (2011). Use of Surveillance, Epidemiology, and end results-Medicare Data to conduct case-control studies of cancer among the US elderly. Am J Epidemiol.

[10] Stocks T, Bjørge T, Ulmer H (2015). Metabolic risk score and cancer risk: pooled analysis of seven cohorts. Int J Epidemiol.

[11] Thrift AP, Shaheen NJ, Gammon MD (2014). Obesity and risk of esophageal adenocarcinoma and Barrett’s esophagus: a mendelian randomization study. J Natl Cancer Inst.

[12] Xu B, Zhou X, Li X (2017). Diabetes mellitus carries a risk of esophageal cancer: a meta-analysis. Med (Baltim).

[13] Iyer PG, Borah BJ, Heien HC (2013). Association of Barrett’s esophagus with type II diabetes Mellitus: results from a large population-based case-control study. Clin Gastroenterol Hepatol.

[14] Wang J, Zhuge J, Feng D (2022). Mendelian randomization study of circulating lipids and biliary tract cancer among East asians. BMC Cancer.

[15] Nowak C, Ärnlöv J (2018). A mendelian randomization study of the effects of blood lipids on breast cancer risk. Nat Commun.

[16] Sako A, Kitayama J, Kaisaki S (2004). Hyperlipidemia is a risk factor for lymphatic metastasis in superficial esophageal carcinoma. Cancer Lett.

[17] Xie SH, Rabbani S, Ness-Jensen E (2020). Circulating levels of inflammatory and metabolic biomarkers and risk of esophageal adenocarcinoma and Barrett Esophagus: systematic review and Meta-analysis. Cancer Epidemiol Biomarkers Prev.

[18] Emdin CA, Khera AV, Kathiresan S (2017). Mendelian randomization. JAMA.

[19] Davey Smith G, Hemani G (2014). Mendelian randomization: genetic anchors for causal inference in epidemiological studies. Hum Mol Genet.

[20] Willer CJ, Schmidt EM, Sengupta S (2013). Discovery and refinement of loci associated with lipid levels. Nat Genet.

[21] Klimentidis YC, Arora A, Newell M (2020). Phenotypic and Genetic Characterization of Lower LDL Cholesterol and increased type 2 diabetes risk in the UK Biobank. Diabetes.

[22] Ong JS, An J, Han X (2022). Multitrait genetic association analysis identifies 50 new risk loci for gastro-oesophageal reflux, seven new loci for Barrett’s oesophagus and provides insights into clinical heterogeneity in reflux diagnosis. Gut.

[23] Eusebi LH, Cirota GG, Zagari RM (2021). Global prevalence of Barrett’s oesophagus and oesophageal cancer in individuals with gastro-oesophageal reflux: a systematic review and meta-analysis. Gut.

[24] Burgess S, Thompson SG. Multivariable mendelian randomization: the use of pleiotropic genetic variants to estimate causal effects. Am J Epidemiol. 2015;181:251–60. 10.1093/aje/kwu283 PMC4325677 25632051

[25] Cao H, Baranova A, Wei X et al. Bidirectional causal associations between type 2 diabetes and COVID-19. J Med Virol. 2022. 10.1002/jmv.28100 PMC9538258 36029131

[26] Bowden J, Davey Smith G, Burgess S (2015). Mendelian randomization with invalid instruments: effect estimation and bias detection through Egger regression. Int J Epidemiol.

[27] Verbanck M, Chen CY, Neale B (2018). Detection of widespread horizontal pleiotropy in causal relationships inferred from mendelian randomization between complex traits and diseases. Nat Genet.

[28] Bowden J, Del Greco MF, Minelli C (2019). Improving the accuracy of two-sample summary-data mendelian randomization: moving beyond the NOME assumption. Int J Epidemiol.

[29] Palmer TM, Lawlor DA, Harbord RM (2012). Using multiple genetic variants as instrumental variables for modifiable risk factors. Stat Methods Med Res.

[30] Shim H, Chasman DI, Smith JD (2015). A multivariate genome-wide association analysis of 10 LDL subfractions, and their response to statin treatment, in 1868 caucasians. PLoS ONE.

[31] Brion MJ, Shakhbazov K, Visscher PM (2013). Calculating statistical power in mendelian randomization studies. Int J Epidemiol.

[32] Hennig B, Toborek M, McClain CJ (2001). High-energy diets, fatty acids and endothelial cell function: implications for atherosclerosis. J Am Coll Nutr.

[33] Huang MC, Graeler M, Shankar G (2002). Lysophospholipid mediators of immunity and neoplasia. Biochim Biophys Acta.

[34] Liu S, Jiang H, Min L (2021). Lysophosphatidic acid mediated PI3K/Akt activation contributed to esophageal squamous cell cancer progression. Carcinogenesis.

[35] Baranova A, Song Y, Cao H (2023). Causal associations between basal metabolic rate and COVID-19. Diabetes.

