# Supplementary material for: The causal associations of circulating lipids with Barrett’s Esophagus and Esophageal Cancer: a bi-directional, two sample mendelian randomization analysis
Source: Hum Genomics. 2024 Apr 16;18:37. doi: 10.1186/s40246-024-00608-6 (PMC11020202; doi:10.1186/s40246-024-00608-6)

**Supplementary material**

**Supplementary tables**

**Table S1. The genetic instruments of triglyceride used in Mendelian analysis.**

| **SNP** | **Chr** | **Position** | **Effect_allele** | **Other_allele** | **Beta** | **Se** | **Beta.BE** | **Se.BE** | **Beta.EC** | **Se.EC** | **R^2^** | **F** |
| --- | --- | --- | --- | --- | --- | --- | --- | --- | --- | --- | --- | --- |
| rs10401969 | 19 | 19407718 | C | T | -0.121 | 0.0065 | 0.0433 | 0.1319 | 0.2363 | 0.191 | 0.0041 | 393 |
| rs10440120 | 3 | 12486964 | A | C | -0.0306 | 0.0044 | -0.0179 | 0.0799 | -0.0865 | 0.1152 | 0.0004 | 40 |
| rs10501321 | 11 | 47294626 | C | T | -0.0216 | 0.0035 | 0.0048 | 0.0687 | 0.053 | 0.0991 | 0.0007 | 68 |
| rs10790162 | 11 | 116639104 | G | A | -0.2305 | 0.0065 | -0.1458 | 0.1191 | -0.1188 | 0.1717 | 0.0037 | 353 |
| rs11057408 | 12 | 124464836 | T | G | -0.0258 | 0.0035 | -0.0597 | 0.0706 | 0.0149 | 0.1018 | 0.0007 | 64 |
| rs11613352 | 12 | 57792580 | T | C | -0.028 | 0.0039 | -0.0348 | 0.0757 | -0.0968 | 0.1094 | 0.0119 | 1140 |
| rs11820504 | 11 | 116529442 | C | T | 0.0604 | 0.0044 | 0.0082 | 0.0702 | -0.0358 | 0.1012 | 0.0005 | 48 |
| rs11974409 | 7 | 72989390 | G | A | -0.0899 | 0.0042 | 0.0067 | 0.0837 | 0.1381 | 0.121 | 0.0006 | 54 |
| rs1260326 | 2 | 27730940 | C | T | -0.1148 | 0.0034 | -0.1147 | 0.0677 | 0.0153 | 0.0976 | 0.0009 | 88 |
| rs12676857 | 8 | 18266572 | C | T | 0.0332 | 0.0046 | -0.0186 | 0.0833 | 0.0455 | 0.12 | 0.0008 | 74 |
| rs12678919 | 8 | 19844222 | G | A | -0.1702 | 0.0056 | -0.0557 | 0.1139 | 0.093 | 0.1645 | 0.0007 | 67 |
| rs12748152 | 1 | 27138393 | T | C | 0.0372 | 0.0059 | -0.1064 | 0.1222 | 0.2321 | 0.1757 | 0.0004 | 41 |
| rs13389219 | 2 | 165528876 | T | C | -0.0271 | 0.0034 | 0.0405 | 0.0677 | -0.1399 | 0.0978 | 0.0007 | 68 |
| rs16948098 | 15 | 44219607 | A | G | 0.08 | 0.0089 | 0.3621 | 0.2426 | -0.6505 | 0.3526 | 0.0004 | 41 |
| rs174535 | 11 | 61551356 | C | T | 0.047 | 0.0034 | 0.1176 | 0.0654 | 0.1103 | 0.0944 | 0.0010 | 94 |
| rs1832007 | 10 | 5254847 | G | A | -0.0327 | 0.0047 | -0.0546 | 0.0923 | -0.0728 | 0.1335 | 0.0003 | 32 |
| rs2043085 | 15 | 58680954 | C | T | -0.0327 | 0.0034 | -0.0658 | 0.0653 | -0.0281 | 0.0942 | 0.0005 | 47 |
| rs2068888 | 10 | 94839642 | A | G | -0.0241 | 0.0034 | 0.0586 | 0.0645 | -0.007 | 0.0931 | 0.0003 | 32 |
| rs2239520 | 6 | 31088922 | A | G | -0.0236 | 0.0037 | -0.096 | 0.0701 | 0.0138 | 0.1016 | 0.0004 | 36 |
| rs2247056 | 6 | 31265490 | C | T | 0.0378 | 0.0039 | -0.1045 | 0.0811 | -0.0568 | 0.1186 | 0.0048 | 458 |
| rs2250802 | 10 | 113921354 | A | G | 0.023 | 0.0037 | 0.0598 | 0.0694 | -0.0679 | 0.1 | 0.0005 | 51 |
| rs247616 | 16 | 56990716 | T | C | -0.0393 | 0.0037 | -0.0502 | 0.0718 | 0.1294 | 0.1038 | 0.0004 | 34 |
| rs2665357 | 6 | 160848167 | C | A | 0.0212 | 0.0033 | 0.101 | 0.0644 | 0.0857 | 0.0931 | 0.0006 | 52 |
| rs287621 | 7 | 130435181 | C | T | -0.0222 | 0.0037 | -0.0192 | 0.0715 | -0.0797 | 0.1029 | 0.0097 | 924 |
| rs2972146 | 2 | 227100698 | T | G | 0.0281 | 0.0034 | 0.0301 | 0.067 | 0.0716 | 0.0966 | 0.0005 | 48 |
| rs3198697 | 16 | 15129940 | T | C | -0.0198 | 0.0034 | -0.0356 | 0.0654 | 0.1182 | 0.0945 | 0.0005 | 50 |
| rs3760627 | 19 | 45457180 | C | T | 0.0189 | 0.0034 | -0.0139 | 0.0643 | 0.05 | 0.0928 | 0.0004 | 39 |
| rs3761445 | 22 | 38595411 | A | G | 0.0232 | 0.0034 | 0.054 | 0.0659 | 0.0813 | 0.0951 | 0.0020 | 188 |
| rs38855 | 7 | 116358044 | G | A | -0.0187 | 0.0033 | -0.0227 | 0.0648 | -0.0811 | 0.0936 | 0.0020 | 191 |
| rs439401 | 19 | 45414451 | C | T | 0.0659 | 0.0038 | -0.0378 | 0.0709 | 0.0174 | 0.1026 | 0.0004 | 38 |
| rs442177 | 4 | 88030261 | T | G | 0.0309 | 0.0033 | -0.0142 | 0.0646 | 0.0537 | 0.0931 | 0.0131 | 1258 |
| rs4587594 | 1 | 63133930 | A | G | -0.0694 | 0.0035 | -0.0165 | 0.0728 | 0.0867 | 0.1054 | 0.0005 | 52 |
| rs4719841 | 7 | 25997536 | G | A | 0.0232 | 0.0034 | 0.1214 | 0.0668 | 0.0547 | 0.0963 | 0.0006 | 54 |
| rs4738684 | 8 | 59406334 | G | A | -0.0205 | 0.0035 | 0.031 | 0.0659 | 0.0129 | 0.0952 | 0.0009 | 81 |
| rs4810479 | 20 | 44545048 | T | C | -0.0474 | 0.0038 | 0.0784 | 0.0741 | -0.1316 | 0.1067 | 0.0010 | 92 |
| rs588136 | 15 | 58730498 | T | C | -0.0495 | 0.0041 | -0.0982 | 0.0764 | 0.0361 | 0.1104 | 0.0015 | 146 |
| rs6029143 | 20 | 39118662 | T | C | -0.0388 | 0.0071 | -0.244 | 0.1779 | 0.2436 | 0.256 | 0.0004 | 34 |
| rs634869 | 6 | 139831757 | C | T | -0.0272 | 0.0033 | -0.0348 | 0.0646 | -0.0489 | 0.093 | 0.0012 | 113 |
| rs676210 | 2 | 21231524 | A | G | -0.0733 | 0.0039 | -0.055 | 0.073 | 0.0136 | 0.1055 | 0.0004 | 36 |
| rs6831256 | 4 | 3473139 | G | A | 0.0258 | 0.0035 | 0.0375 | 0.0677 | -0.0442 | 0.0975 | 0.0005 | 43 |
| rs6882076 | 5 | 156390297 | C | T | 0.0286 | 0.0035 | 0.0724 | 0.0685 | -0.1307 | 0.099 | 0.0036 | 347 |
| rs6995541 | 8 | 10671260 | G | A | 0.0265 | 0.0037 | -0.024 | 0.0749 | -0.0308 | 0.1081 | 0.0004 | 39 |
| rs719726 | 6 | 127414801 | T | C | 0.0199 | 0.0035 | 0.0173 | 0.0654 | 0.021 | 0.0942 | 0.0032 | 301 |
| rs7248104 | 19 | 7224431 | A | G | -0.0222 | 0.0034 | -0.0546 | 0.0651 | -0.1395 | 0.094 | 0.0003 | 31 |
| rs731839 | 19 | 33899065 | A | G | -0.0224 | 0.0036 | 0.064 | 0.0679 | -0.0172 | 0.0979 | 0.0016 | 156 |
| rs8077889 | 17 | 41878166 | C | A | 0.0252 | 0.0042 | 0.011 | 0.0787 | 0.0537 | 0.1136 | 0.0003 | 30 |
| rs9686661 | 5 | 55861786 | T | C | 0.0379 | 0.0044 | 0.0225 | 0.0951 | 0.1172 | 0.1367 | 0.0005 | 47 |

**Table S2. The genetic instruments of HDL cholesterol used in Mendelian analys**is.

| **SNP** | **Chr** | **Pos** | **Effect_allele** | **Other_allele** | **Beta** | **Se** | **Beta.BE** | **Se.BE** | **Beta.EC** | **Se.EC** | **R^2^** | **F** |
| --- | --- | --- | --- | --- | --- | --- | --- | --- | --- | --- | --- | --- |
| rs10019888 | 4 | 26062990 | G | A | -0.027 | 0.0046 | -0.1277 | 0.0948 | -0.2757 | 0.1362 | 0.0004 | 34 |
| rs10087900 | 8 | 144303418 | A | G | -0.0231 | 0.0036 | -0.0241 | 0.0664 | -0.0475 | 0.0959 | 0.0004 | 41 |
| rs10282707 | 7 | 17911038 | T | C | -0.025 | 0.0035 | -0.0916 | 0.0689 | -0.1817 | 0.0993 | 0.0005 | 51 |
| rs10468017 | 15 | 58678512 | T | C | 0.1179 | 0.0038 | 0.1011 | 0.0684 | 0.0144 | 0.0988 | 0.0101 | 963 |
| rs10808546 | 8 | 126495818 | T | C | 0.0409 | 0.0034 | -0.0463 | 0.065 | 0.0274 | 0.0937 | 0.0015 | 145 |
| rs11045163 | 12 | 20463526 | G | A | 0.0217 | 0.0035 | 0.0156 | 0.0663 | 0.1217 | 0.0953 | 0.0004 | 38 |
| rs11065987 | 12 | 112072424 | G | A | -0.0222 | 0.0035 | -0.0629 | 0.0663 | -0.0985 | 0.0957 | 0.0004 | 40 |
| rs11789603 | 9 | 107647019 | T | C | 0.06 | 0.006 | -0.0761 | 0.1206 | 0.1598 | 0.1753 | 0.0011 | 100 |
| rs12133576 | 1 | 93816400 | G | A | -0.0243 | 0.0035 | -0.0425 | 0.0678 | 0.0654 | 0.0977 | 0.0005 | 48 |
| rs12145743 | 1 | 156700651 | G | T | 0.0203 | 0.0036 | 0.0087 | 0.0679 | 0.0151 | 0.0981 | 0.0003 | 32 |
| rs12286037 | 11 | 116652207 | T | C | -0.1052 | 0.007 | 0.0545 | 0.1312 | 0.0733 | 0.1892 | 0.0024 | 226 |
| rs12328675 | 2 | 165540800 | C | T | 0.0447 | 0.0052 | 0.0273 | 0.1084 | -0.1745 | 0.1567 | 0.0008 | 74 |
| rs12412743 | 10 | 114045333 | T | C | -0.0291 | 0.0045 | -0.0994 | 0.0884 | -0.1498 | 0.1279 | 0.0004 | 42 |
| rs12740374 | 1 | 109817590 | T | G | 0.0343 | 0.0041 | -0.1444 | 0.0786 | 0.1275 | 0.1137 | 0.0007 | 70 |
| rs12748152 | 1 | 27138393 | T | C | -0.0506 | 0.0062 | -0.1064 | 0.1222 | 0.2321 | 0.1757 | 0.0007 | 67 |
| rs13099479 | 3 | 52677478 | A | G | 0.036 | 0.0062 | 0.1469 | 0.1204 | 0.1068 | 0.1749 | 0.0004 | 34 |
| rs16842 | 8 | 19968929 | C | T | -0.03 | 0.0038 | 0.1736 | 0.075 | 0.1086 | 0.1087 | 0.0007 | 62 |
| rs1689797 | 1 | 182150978 | A | C | -0.0358 | 0.0036 | 0.0605 | 0.0708 | 0.0783 | 0.1024 | 0.0010 | 99 |
| rs16942887 | 16 | 67928042 | A | G | 0.0831 | 0.0051 | 0.1756 | 0.0906 | 0.0666 | 0.1308 | 0.0028 | 265 |
| rs17145738 | 7 | 72982874 | T | C | 0.0408 | 0.0053 | -0.1054 | 0.0963 | 0.1422 | 0.1389 | 0.0006 | 59 |
| rs17173637 | 7 | 150529449 | C | T | -0.0363 | 0.0057 | -7.00E-04 | 0.0926 | 0.116 | 0.1333 | 0.0004 | 41 |
| rs174535 | 11 | 61551356 | C | T | -0.0392 | 0.0035 | 0.1176 | 0.0654 | 0.1103 | 0.0944 | 0.0013 | 125 |
| rs181362 | 22 | 21932068 | T | C | -0.0379 | 0.0042 | 0.047 | 0.0699 | -0.1257 | 0.1006 | 0.0009 | 81 |
| rs1883025 | 9 | 107664301 | T | C | -0.0698 | 0.0041 | 0.1235 | 0.0812 | -0.0366 | 0.1177 | 0.0031 | 290 |
| rs1980493 | 6 | 32363215 | C | T | -0.0318 | 0.0048 | -0.0526 | 0.0984 | -0.1322 | 0.1447 | 0.0005 | 44 |
| rs2066714 | 9 | 107586753 | C | T | 0.0453 | 0.0071 | 0.0487 | 0.111 | 0.0958 | 0.1601 | 0.0004 | 41 |
| rs2160669 | 11 | 116647607 | T | C | 0.0944 | 0.0069 | -0.1239 | 0.1175 | -0.0838 | 0.1693 | 0.0020 | 187 |
| rs2241210 | 12 | 109950144 | G | A | 0.0332 | 0.0035 | 0.0381 | 0.0645 | -0.0099 | 0.093 | 0.0010 | 90 |
| rs2255141 | 10 | 113933886 | G | A | -0.0337 | 0.0037 | 0.0625 | 0.0694 | -0.0698 | 0.1 | 0.0009 | 83 |
| rs2278236 | 19 | 8431581 | A | G | 0.0331 | 0.0035 | 0.0037 | 0.0645 | -0.0284 | 0.0931 | 0.0009 | 89 |
| rs2288911 | 19 | 45449284 | G | T | 0.0302 | 0.0036 | -0.006 | 0.0646 | -0.0091 | 0.0931 | 0.0007 | 70 |
| rs2454722 | 12 | 123171218 | G | A | 0.0351 | 0.0044 | -0.066 | 0.0869 | 0.016 | 0.1257 | 0.0007 | 64 |
| rs247616 | 16 | 56990716 | T | C | 0.243 | 0.0038 | -0.0502 | 0.0718 | 0.1294 | 0.1038 | 0.0414 | 4089 |
| rs2602836 | 4 | 100014805 | G | A | -0.0192 | 0.0034 | 1.00E-04 | 0.0648 | 0.07 | 0.0934 | 0.0003 | 32 |
| rs2606736 | 3 | 11400249 | T | C | -0.0246 | 0.0043 | 0.0675 | 0.0653 | 0.1383 | 0.0942 | 0.0003 | 33 |
| rs2642438 | 1 | 220970028 | G | A | 0.0303 | 0.0039 | -0.011 | 0.0715 | -0.1558 | 0.1033 | 0.0006 | 60 |
| rs289745 | 16 | 57019532 | A | C | 0.0276 | 0.0041 | -0.044 | 0.0685 | 0.197 | 0.0992 | 0.0005 | 45 |
| rs2925979 | 16 | 81534790 | C | T | 0.0351 | 0.0037 | -0.0073 | 0.069 | 0.0523 | 0.0996 | 0.0010 | 90 |
| rs2972146 | 2 | 227100698 | T | G | -0.0323 | 0.0035 | 0.0301 | 0.067 | 0.0716 | 0.0966 | 0.0009 | 85 |
| rs326214 | 11 | 47298360 | A | G | -0.0609 | 0.0045 | -0.0056 | 0.0687 | -0.0542 | 0.0991 | 0.0019 | 183 |
| rs3741414 | 12 | 57844049 | T | C | 0.0296 | 0.004 | -0.0296 | 0.0758 | -0.0999 | 0.1096 | 0.0006 | 55 |
| rs3822072 | 4 | 89741269 | A | G | -0.0251 | 0.0034 | -0.1326 | 0.0644 | 0.0916 | 0.0929 | 0.0006 | 54 |
| rs3829502 | 16 | 56896730 | A | G | 0.0482 | 0.0051 | -0.0832 | 0.0658 | -0.0216 | 0.095 | 0.0009 | 89 |
| rs3936511 | 5 | 55860781 | G | A | -0.0308 | 0.0046 | 0.0257 | 0.0949 | 0.0309 | 0.1368 | 0.0005 | 45 |
| rs3996352 | 7 | 130444934 | G | A | 0.0296 | 0.0034 | 0.0129 | 0.0646 | 0.058 | 0.0932 | 0.0008 | 76 |
| rs4148005 | 17 | 66882466 | G | T | -0.0283 | 0.0036 | -0.0332 | 0.0705 | 0.2232 | 0.1016 | 0.0007 | 62 |
| rs4240624 | 8 | 9184231 | A | G | 0.0818 | 0.0058 | 0.26 | 0.0972 | 0.2552 | 0.14 | 0.0021 | 199 |
| rs424346 | 15 | 59010962 | T | C | 0.0679 | 0.0113 | 0.1623 | 0.1726 | -0.2453 | 0.2478 | 0.0004 | 36 |
| rs4379922 | 12 | 125351116 | C | T | 0.0247 | 0.0036 | -0.0184 | 0.067 | -0.0862 | 0.0966 | 0.0005 | 47 |
| rs4465830 | 20 | 44585420 | G | A | -0.0597 | 0.0044 | -0.1387 | 0.0902 | 0.2725 | 0.1298 | 0.0019 | 184 |
| rs4650994 | 1 | 178515312 | A | G | -0.021 | 0.0034 | 0.073 | 0.0647 | -0.0446 | 0.0934 | 0.0004 | 38 |
| rs4846914 | 1 | 230295691 | A | G | 0.0479 | 0.0034 | -0.0123 | 0.0649 | -0.2104 | 0.0939 | 0.0021 | 198 |
| rs4917014 | 7 | 50305863 | G | T | 0.0222 | 0.0036 | 0.0921 | 0.0701 | -0.2065 | 0.1011 | 0.0004 | 38 |
| rs492571 | 15 | 44211273 | C | T | -0.0663 | 0.009 | 0.3697 | 0.2431 | -0.6482 | 0.3532 | 0.0006 | 54 |
| rs4939883 | 18 | 47167214 | C | T | 0.0799 | 0.0045 | 0.1279 | 0.0843 | 0.1443 | 0.1221 | 0.0033 | 315 |
| rs4969178 | 17 | 76388202 | G | A | 0.0263 | 0.0035 | -0.0177 | 0.0677 | -0.0679 | 0.0979 | 0.0006 | 56 |
| rs4983559 | 14 | 105277209 | A | G | -0.0197 | 0.0036 | -0.0538 | 0.0665 | 0.0506 | 0.096 | 0.0003 | 30 |
| rs499974 | 11 | 75455021 | A | C | -0.0263 | 0.0044 | 0.008 | 0.0766 | 0.1321 | 0.1105 | 0.0004 | 36 |
| rs6031587 | 20 | 43038249 | T | C | -0.0488 | 0.0074 | -0.0434 | 0.1246 | -0.071 | 0.1794 | 0.0005 | 43 |
| rs633695 | 15 | 58725839 | G | A | 0.0885 | 0.0054 | 0.0786 | 0.07 | -0.0274 | 0.1012 | 0.0028 | 269 |
| rs634869 | 6 | 139831757 | C | T | 0.0234 | 0.0034 | -0.0348 | 0.0646 | -0.0489 | 0.093 | 0.0005 | 47 |
| rs6509859 | 19 | 54762408 | T | C | -0.0328 | 0.0046 | 0.0493 | 0.0749 | -0.1726 | 0.1086 | 0.0005 | 51 |
| rs676210 | 2 | 21231524 | A | G | 0.066 | 0.004 | -0.055 | 0.073 | 0.0136 | 0.1055 | 0.0029 | 272 |
| rs6805251 | 3 | 119560606 | C | T | -0.02 | 0.0035 | -0.0467 | 0.0656 | -0.0216 | 0.0948 | 0.0003 | 33 |
| rs686030 | 9 | 15304782 | A | C | 0.055 | 0.0049 | 0.1753 | 0.0986 | -0.0494 | 0.1434 | 0.0013 | 126 |
| rs702485 | 7 | 6449272 | G | A | 0.0243 | 0.0034 | -0.0352 | 0.0645 | -0.1277 | 0.093 | 0.0005 | 51 |
| rs7306660 | 12 | 125327384 | A | G | -0.0345 | 0.0036 | 0.0271 | 0.0693 | -0.0336 | 0.1001 | 0.0010 | 92 |
| rs731839 | 19 | 33899065 | A | G | 0.022 | 0.0037 | 0.064 | 0.0679 | -0.0172 | 0.0979 | 0.0004 | 35 |
| rs737337 | 19 | 11347493 | C | T | -0.0565 | 0.0061 | -0.0018 | 0.1112 | -0.1413 | 0.1611 | 0.0009 | 86 |
| rs765548 | 8 | 19866594 | T | C | 0.1065 | 0.0038 | 0.0232 | 0.0742 | 0.0956 | 0.1073 | 0.0082 | 785 |
| rs838876 | 12 | 125259888 | G | A | -0.0493 | 0.0039 | -0.1059 | 0.0656 | -0.0141 | 0.0948 | 0.0017 | 160 |
| rs931992 | 17 | 37821435 | T | G | 0.034 | 0.0036 | -0.043 | 0.0693 | 0.006 | 0.1001 | 0.0009 | 89 |
| rs9457931 | 6 | 160929904 | G | A | -0.0552 | 0.0073 | -0.0265 | 0.1011 | -0.1346 | 0.146 | 0.0006 | 57 |
| rs970548 | 10 | 46013277 | C | A | 0.0258 | 0.0039 | -0.0686 | 0.0714 | 0.0914 | 0.1031 | 0.0005 | 44 |

**Table S3. The genetic instruments of LDL cholesterol used in Mendelian analysis.**

| **SNP** | **Chr** | **Pos** | **Effect_allele** | **Other_allele** | **Beta** | **Se** | **Beta.BE** | **Se.BE** | **Beta.EC** | **Se.EC** | **R^2^** | **F** |
| --- | --- | --- | --- | --- | --- | --- | --- | --- | --- | --- | --- | --- |
| rs10021804 | 4 | 110942596 | G | A | -0.011471 | 0.00197637 | -0.0576 | 0.0665 | -0.1018 | 0.0961 | 0.0001 | 34 |
| rs10025454 | 4 | 39486969 | A | T | -0.0121934 | 0.00204198 | -0.0447 | 0.066 | -0.0457 | 0.0953 | 0.0001 | 36 |
| rs10076475 | 5 | 156422231 | A | G | 0.01221 | 0.00193344 | 0.0519 | 0.0654 | -0.1785 | 0.0945 | 0.0001 | 40 |
| rs10145740 | 14 | 74234294 | T | C | -0.0183362 | 0.00224034 | -0.0564 | 0.0818 | 0.1078 | 0.1182 | 0.0002 | 67 |
| rs1016102 | 8 | 87562786 | C | T | 0.0132695 | 0.00228578 | 0.0668 | 0.0738 | 0.0822 | 0.1065 | 0.0001 | 34 |
| rs10184004 | 2 | 165508389 | T | C | -0.0183546 | 0.00192292 | 0.0329 | 0.0672 | -0.143 | 0.0971 | 0.0002 | 91 |
| rs10272002 | 7 | 1047615 | G | A | -0.0210319 | 0.00234079 | -0.054 | 0.0736 | -0.1006 | 0.1062 | 0.0002 | 81 |
| rs10438978 | 18 | 47158186 | C | T | 0.0248331 | 0.0025037 | 0.1402 | 0.084 | 0.1611 | 0.1217 | 0.0002 | 98 |
| rs10445374 | 17 | 45662383 | C | T | -0.0359766 | 0.00191809 | -0.0775 | 0.0645 | -0.1082 | 0.0931 | 0.0008 | 352 |
| rs10448340 | 9 | 139320069 | G | T | -0.0166194 | 0.00205322 | -0.0223 | 0.07 | -0.0827 | 0.101 | 0.0002 | 66 |
| rs10455872 | 6 | 161010118 | G | A | 0.135534 | 0.00353711 | 0.2544 | 0.1534 | 0.1327 | 0.2218 | 0.0034 | 1468 |
| rs10500834 | 11 | 18638712 | T | A | 0.0241684 | 0.00217547 | 0.0042 | 0.0662 | 0.0567 | 0.0955 | 0.0003 | 123 |
| rs10794579 | 10 | 124686656 | C | T | 0.0201777 | 0.00193933 | -0.1032 | 0.0667 | -0.1115 | 0.0962 | 0.0003 | 108 |
| rs10795464 | 10 | 17255095 | A | G | 0.0144189 | 0.00197467 | -0.0779 | 0.0656 | 0.0305 | 0.0948 | 0.0001 | 53 |
| rs10808709 | 8 | 61494991 | C | G | 0.0142412 | 0.00198143 | -0.0726 | 0.0656 | -0.0841 | 0.0946 | 0.0001 | 52 |
| rs1081105 | 19 | 45412955 | C | A | 0.221489 | 0.00565339 | 0.0534 | 0.2237 | 0.1298 | 0.3249 | 0.0035 | 1535 |
| rs10817718 | 9 | 117925945 | A | G | -0.0123176 | 0.00210538 | 0.0893 | 0.0734 | -0.1458 | 0.106 | 0.0001 | 34 |
| rs10843391 | 12 | 29508642 | G | A | -0.0117868 | 0.00212478 | -0.0376 | 0.0705 | -0.0467 | 0.1018 | 0.0001 | 31 |
| rs10846740 | 12 | 125303282 | C | T | -0.0309308 | 0.00279237 | -0.0253 | 0.0919 | 0.0676 | 0.1331 | 0.0003 | 123 |
| rs10869598 | 9 | 78214452 | C | T | 0.016609 | 0.00209097 | 0.0158 | 0.0651 | 0.0041 | 0.0941 | 0.0001 | 63 |
| rs10877955 | 12 | 40421117 | A | G | 0.028411 | 0.00393318 | -0.0557 | 0.1795 | -0.3646 | 0.259 | 0.0001 | 52 |
| rs10910476 | 1 | 234734956 | T | C | 0.0156095 | 0.0019078 | -0.0496 | 0.0644 | 0.0556 | 0.0932 | 0.0002 | 67 |
| rs10910522 | 1 | 235011725 | A | G | -0.0117792 | 0.00191916 | 0.0796 | 0.0658 | -0.0839 | 0.0948 | 0.0001 | 38 |
| rs10930590 | 2 | 174253799 | A | G | 0.0111478 | 0.00201374 | -0.0198 | 0.0722 | 0.1849 | 0.1044 | 0.0001 | 31 |
| rs10953260 | 7 | 98033492 | C | T | 0.0228986 | 0.00248097 | -0.0016 | 0.0898 | 0.0473 | 0.1299 | 0.0002 | 85 |
| rs10953298 | 7 | 100216773 | T | C | -0.0229706 | 0.00226348 | -0.0033 | 0.0749 | -0.0189 | 0.1082 | 0.0002 | 103 |
| rs11057397 | 12 | 124419728 | T | C | -0.0186053 | 0.00202681 | -0.0447 | 0.0703 | -0.0032 | 0.1014 | 0.0002 | 84 |
| rs11085721 | 19 | 10317976 | C | G | -0.016179 | 0.0025225 | -0.0201 | 0.1098 | 0.0965 | 0.158 | 0.0001 | 41 |
| rs111278137 | 19 | 45215081 | A | G | -0.112997 | 0.00659977 | -0.0927 | 0.3225 | 0.6611 | 0.462 | 0.0007 | 293 |
| rs111371088 | 20 | 39128165 | C | T | -0.0353733 | 0.00569135 | -0.1747 | 0.3343 | -0.2281 | 0.4751 | 0.0001 | 39 |
| rs11159071 | 14 | 74682373 | T | C | -0.0107128 | 0.00193087 | 0.1932 | 0.065 | -0.0711 | 0.0937 | 0.0001 | 31 |
| rs111928762 | 1 | 55695535 | G | A | 0.0716397 | 0.00486008 | 0.1351 | 0.1379 | -2.00E-04 | 0.1987 | 0.0005 | 217 |
| rs111990272 | 11 | 122547206 | A | G | 0.0140366 | 0.00221541 | 0.0958 | 0.0736 | -0.0793 | 0.1066 | 0.0001 | 40 |
| rs112201728 | 6 | 160551486 | T | C | 0.0671186 | 0.00375323 | -0.0455 | 0.1379 | -0.0461 | 0.1982 | 0.0007 | 320 |
| rs11226108 | 11 | 103870755 | C | G | -0.0194011 | 0.00243145 | 0.171 | 0.0834 | -0.0505 | 0.121 | 0.0001 | 64 |
| rs11231711 | 11 | 63929215 | A | G | 0.0262637 | 0.00411844 | 0.1615 | 0.1114 | 0.1378 | 0.1606 | 0.0001 | 41 |
| rs112679104 | 13 | 74706951 | T | C | -0.0475502 | 0.00768498 | 0.3227 | 0.184 | -0.0434 | 0.2668 | 0.0001 | 38 |
| rs112693563 | 1 | 45237714 | C | T | 0.0152164 | 0.00234105 | -0.0237 | 0.0767 | 0.1323 | 0.1109 | 0.0001 | 42 |
| rs112811239 | 5 | 156689921 | C | T | -0.0135468 | 0.00233135 | -0.0814 | 0.0747 | 0.1146 | 0.1078 | 0.0001 | 34 |
| rs113111921 | 7 | 100648875 | A | C | -0.0183685 | 0.0029188 | -0.1678 | 0.1018 | -0.1489 | 0.1512 | 0.0001 | 40 |
| rs1133790 | 7 | 99632982 | C | T | -0.0114272 | 0.00207336 | 0.0501 | 0.0688 | -0.1373 | 0.0992 | 0.0001 | 30 |
| rs1134027 | 8 | 145051414 | A | G | 0.0243881 | 0.00199538 | -0.0303 | 0.0666 | -0.2407 | 0.0963 | 0.0003 | 149 |
| rs114165349 | 1 | 27021913 | C | G | 0.0914981 | 0.00630213 | -0.1315 | 0.1737 | 0.0756 | 0.2494 | 0.0005 | 211 |
| rs114756490 | 4 | 155476627 | A | G | 0.0637655 | 0.00868399 | -0.388 | 0.4671 | -0.9858 | 0.6719 | 0.0001 | 54 |
| rs114783000 | 4 | 126402260 | G | A | -0.0255399 | 0.00461203 | -0.3119 | 0.1271 | -0.3081 | 0.1831 | 0.0001 | 31 |
| rs115383270 | 1 | 161531340 | A | G | 0.0274859 | 0.00365226 | -0.1837 | 0.1374 | 0.3407 | 0.1972 | 0.0001 | 57 |
| rs11568318 | 2 | 234665498 | A | C | 0.0336663 | 0.00379062 | 0.3986 | 0.1862 | 0.2223 | 0.2722 | 0.0002 | 79 |
| rs11591147 | 1 | 55505647 | T | G | -0.457849 | 0.0071963 | -0.0927 | 0.1772 | 0.1499 | 0.2543 | 0.0093 | 4048 |
| rs11601507 | 11 | 5701074 | A | C | 0.0493409 | 0.00371368 | 0.0738 | 0.12 | -0.12 | 0.173 | 0.0004 | 177 |
| rs11621792 | 14 | 24871926 | T | C | 0.0267004 | 0.00194171 | 0.0514 | 0.0667 | -0.0272 | 0.0964 | 0.0004 | 189 |
| rs11641811 | 16 | 71635836 | A | C | 0.0293316 | 0.00191805 | 0.0237 | 0.0655 | 0.1041 | 0.0948 | 0.0005 | 234 |
| rs11646091 | 16 | 70825649 | C | T | -0.0342311 | 0.00479134 | 0.0304 | 0.1251 | 0.2651 | 0.1795 | 0.0001 | 51 |
| rs11652501 | 17 | 80524546 | C | G | -0.0115693 | 0.00199198 | 0.0715 | 0.0661 | -0.0125 | 0.0953 | 0.0001 | 34 |
| rs1169288 | 12 | 121416650 | C | A | 0.0383025 | 0.00207161 | 0.0309 | 0.0671 | -0.008 | 0.0966 | 0.0008 | 342 |
| rs117733303 | 6 | 160922870 | G | A | 0.155275 | 0.00707981 | 0.1569 | 0.3036 | -0.4504 | 0.4358 | 0.0011 | 481 |
| rs11789603 | 9 | 107647019 | T | C | 0.0309585 | 0.00308951 | -0.0761 | 0.1206 | 0.1598 | 0.1753 | 0.0002 | 100 |
| rs11846704 | 14 | 35186694 | T | C | -0.0128621 | 0.00217476 | -0.0669 | 0.0716 | -0.1152 | 0.1035 | 0.0001 | 35 |
| rs11895352 | 2 | 20367135 | T | C | -0.0229815 | 0.00189418 | -0.0345 | 0.0653 | 0.2673 | 0.0946 | 0.0003 | 147 |
| rs11997161 | 8 | 141738587 | C | T | 0.0106387 | 0.00191608 | -0.0399 | 0.0651 | -0.0407 | 0.094 | 0.0001 | 31 |
| rs12027388 | 1 | 26812192 | G | C | -0.017145 | 0.00232306 | -0.025 | 0.0752 | -0.0909 | 0.1086 | 0.0001 | 54 |
| rs12054451 | 3 | 122064369 | G | T | 0.0191916 | 0.00219453 | -0.048 | 0.0762 | -0.019 | 0.1099 | 0.0002 | 76 |
| rs12202204 | 6 | 126686407 | G | A | 0.0139897 | 0.00205564 | 0.0282 | 0.073 | -0.034 | 0.1056 | 0.0001 | 46 |
| rs12212146 | 6 | 161125454 | C | T | -0.0237755 | 0.00373812 | 0.0844 | 0.1338 | 0.169 | 0.1941 | 0.0001 | 40 |
| rs12271333 | 11 | 5690691 | C | A | 0.01835 | 0.00276569 | -0.1584 | 0.1008 | -0.0555 | 0.1452 | 0.0001 | 44 |
| rs12320328 | 12 | 25408464 | G | A | -0.022934 | 0.00345496 | -0.1337 | 0.0911 | 0.1498 | 0.1317 | 0.0001 | 44 |
| rs12445804 | 16 | 11706100 | A | G | 0.0363752 | 0.00367779 | -0.0049 | 0.1038 | -0.1292 | 0.1496 | 0.0002 | 98 |
| rs12453219 | 17 | 76392756 | G | C | -0.0191522 | 0.00271222 | -0.0632 | 0.109 | 0.2308 | 0.1572 | 0.0001 | 50 |
| rs12469822 | 2 | 113830563 | A | G | -0.0112645 | 0.00193914 | -0.0577 | 0.0656 | 0.0514 | 0.0946 | 0.0001 | 34 |
| rs12471768 | 2 | 64928603 | C | T | 0.014803 | 0.00207156 | -0.0334 | 0.0761 | -0.019 | 0.1099 | 0.0001 | 51 |
| rs1250259 | 2 | 216300482 | A | T | 0.0157213 | 0.00214831 | 0.1289 | 0.0788 | -0.2914 | 0.114 | 0.0001 | 54 |
| rs1260326 | 2 | 27730940 | C | T | -0.0529385 | 0.00193189 | -0.1147 | 0.0677 | 0.0153 | 0.0976 | 0.0017 | 751 |
| rs12614487 | 2 | 158434569 | T | C | -0.0276135 | 0.00357121 | 0.2456 | 0.1363 | 0.1463 | 0.1978 | 0.0001 | 60 |
| rs12638256 | 3 | 142655290 | G | A | -0.0155767 | 0.00192518 | -0.1234 | 0.067 | 0.0637 | 0.0968 | 0.0002 | 65 |
| rs12655342 | 5 | 96229542 | G | T | -0.0163171 | 0.00274509 | -0.1491 | 0.0858 | -0.0396 | 0.1236 | 0.0001 | 35 |
| rs12665537 | 6 | 53509452 | G | A | 0.0181891 | 0.00203422 | -0.0138 | 0.0684 | 0.1141 | 0.0987 | 0.0002 | 80 |
| rs12684235 | 9 | 94189602 | A | G | -0.0217118 | 0.00396064 | -0.0648 | 0.1359 | -0.1114 | 0.1957 | 0.0001 | 30 |
| rs12740374 | 1 | 109817590 | T | G | -0.162741 | 0.00227119 | -0.1444 | 0.0786 | 0.1275 | 0.1137 | 0.0118 | 5134 |
| rs12977362 | 19 | 11306078 | T | C | 0.0213492 | 0.00189345 | -0.0174 | 0.0649 | 0.101 | 0.0937 | 0.0003 | 127 |
| rs12985907 | 19 | 41343544 | A | G | -0.0158515 | 0.00220062 | 0.0483 | 0.0732 | 0.0432 | 0.1056 | 0.0001 | 52 |
| rs12986248 | 19 | 47252875 | G | A | -0.0138055 | 0.0018929 | -0.0149 | 0.0658 | 0.0106 | 0.0949 | 0.0001 | 53 |
| rs13076933 | 3 | 12327431 | G | T | -0.0322061 | 0.0021932 | -0.0709 | 0.0725 | 0.0069 | 0.1047 | 0.0005 | 216 |
| rs13108218 | 4 | 3443931 | G | A | -0.0275204 | 0.00198645 | 0.009 | 0.0692 | -0.0146 | 0.0999 | 0.0004 | 192 |
| rs13255048 | 8 | 18276640 | A | G | 0.0332894 | 0.00276766 | -0.0116 | 0.0839 | 0.0225 | 0.1209 | 0.0003 | 145 |
| rs1336455 | 10 | 97838474 | A | G | -0.0116911 | 0.00199733 | 0.093 | 0.0689 | 0.0478 | 0.0996 | 0.0001 | 34 |
| rs1349852 | 4 | 69533217 | C | A | 0.0165743 | 0.00193311 | 0.002 | 0.0651 | -0.1827 | 0.0939 | 0.0002 | 74 |
| rs1362965 | 12 | 51321994 | T | C | 0.0134411 | 0.00198053 | -0.1204 | 0.0672 | -0.0073 | 0.0971 | 0.0001 | 46 |
| rs1375131 | 2 | 135954797 | C | T | 0.0182348 | 0.00218398 | -0.0124 | 0.0659 | -0.0058 | 0.0951 | 0.0002 | 70 |
| rs13796 | 1 | 154245917 | C | T | 0.0163122 | 0.00275844 | 0.1044 | 0.1122 | -0.0878 | 0.1618 | 0.0001 | 35 |
| rs138352 | 22 | 41268925 | G | T | -0.0163988 | 0.00203396 | 0.0653 | 0.0666 | -0.0115 | 0.0961 | 0.0002 | 65 |
| rs141783576 | 6 | 127439897 | C | G | 0.0312637 | 0.00375077 | -0.1476 | 0.119 | 0.1005 | 0.1713 | 0.0002 | 69 |
| rs1434282 | 1 | 199010721 | T | C | 0.0128446 | 0.00212002 | -0.1273 | 0.071 | 8.00E-04 | 0.1024 | 0.0001 | 37 |
| rs143455776 | 5 | 52112850 | C | T | -0.0552804 | 0.00536521 | 0.0756 | 0.22 | -0.0407 | 0.317 | 0.0002 | 106 |
| rs146433259 | 5 | 34713792 | T | C | -0.0577472 | 0.00952965 | 0.3575 | 0.4814 | -0.5732 | 0.7143 | 0.0001 | 37 |
| rs147510296 | 7 | 44895359 | A | G | 0.0379851 | 0.00655185 | 0.2511 | 0.2749 | 0.1517 | 0.4085 | 0.0001 | 34 |
| rs147539187 | 6 | 11839042 | G | C | -0.0311891 | 0.00366627 | -0.0674 | 0.2081 | -0.2468 | 0.3007 | 0.0002 | 72 |
| rs14842 | 22 | 46639457 | T | A | 0.0197196 | 0.00307642 | -0.0602 | 0.1181 | -0.1044 | 0.1706 | 0.0001 | 41 |
| rs1489501 | 11 | 26082951 | C | G | -0.0173911 | 0.00294144 | -0.0992 | 0.133 | 0.0662 | 0.1937 | 0.0001 | 35 |
| rs1497406 | 1 | 16505320 | G | A | 0.0192839 | 0.00190754 | -0.0025 | 0.0689 | -0.1975 | 0.0997 | 0.0002 | 102 |
| rs150474434 | 2 | 118845121 | A | G | -0.0433941 | 0.00315131 | -0.1557 | 0.1202 | -0.0663 | 0.1731 | 0.0004 | 190 |
| rs150783681 | 4 | 74256021 | C | G | 0.0451238 | 0.00674523 | -0.0604 | 0.2495 | 0.0971 | 0.3562 | 0.0001 | 45 |
| rs15285 | 8 | 19824667 | T | C | -0.0284133 | 0.00211509 | 0.0016 | 0.0734 | 0.0704 | 0.106 | 0.0004 | 180 |
| rs1532085 | 15 | 58683366 | G | A | -0.0264105 | 0.00197206 | -0.0595 | 0.0653 | -0.0358 | 0.0942 | 0.0004 | 179 |
| rs1544155 | 6 | 116346394 | A | C | -0.0208176 | 0.001932 | -0.0099 | 0.066 | 0.0369 | 0.0952 | 0.0003 | 116 |
| rs1571790 | 9 | 78729176 | T | A | -0.0174563 | 0.00197637 | -0.0788 | 0.0673 | -0.1579 | 0.0973 | 0.0002 | 78 |
| rs165722 | 22 | 19949013 | T | C | 0.0114996 | 0.00193704 | -0.1194 | 0.0647 | 0.0846 | 0.0934 | 0.0001 | 35 |
| rs16988410 | 22 | 30617585 | C | T | 0.0265452 | 0.0045791 | -0.1114 | 0.0996 | -0.0123 | 0.1441 | 0.0001 | 34 |
| rs17031776 | 2 | 44181351 | T | C | 0.0151406 | 0.00221634 | 0.0554 | 0.077 | 0.0374 | 0.111 | 0.0001 | 47 |
| rs17036085 | 1 | 109834039 | G | A | -0.0774542 | 0.00862011 | -0.2563 | 0.1649 | 0.0208 | 0.2373 | 0.0002 | 81 |
| rs17050272 | 2 | 121306440 | A | G | -0.0260593 | 0.00192093 | -0.0308 | 0.0649 | 0.081 | 0.0937 | 0.0004 | 184 |
| rs17137472 | 7 | 17296072 | T | C | 0.0110657 | 0.00191756 | 0.0089 | 0.0655 | 0.1543 | 0.0945 | 0.0001 | 33 |
| rs174564 | 11 | 61588305 | G | A | -0.0407562 | 0.00200581 | 0.1143 | 0.0654 | 0.1151 | 0.0944 | 0.0010 | 413 |
| rs17476364 | 10 | 71094504 | C | T | -0.033023 | 0.00308568 | 0.2069 | 0.1353 | -0.333 | 0.1975 | 0.0003 | 115 |
| rs17580 | 14 | 94847262 | A | T | 0.0514106 | 0.00450828 | -0.1456 | 0.3378 | -0.0586 | 0.4912 | 0.0003 | 130 |
| rs17712208 | 1 | 214150445 | A | T | 0.0296226 | 0.00511677 | 0.0104 | 0.2386 | -0.1829 | 0.3472 | 0.0001 | 34 |
| rs17875609 | 19 | 45820144 | T | C | -0.0629727 | 0.00624071 | -0.3153 | 0.3123 | -0.5684 | 0.4478 | 0.0002 | 102 |
| rs1800562 | 6 | 26093141 | A | G | -0.0645775 | 0.00358339 | 0.1519 | 0.1695 | 0.0672 | 0.2475 | 0.0008 | 325 |
| rs1800961 | 20 | 43042364 | T | C | -0.0755416 | 0.0055309 | -0.0777 | 0.1543 | 0.144 | 0.2227 | 0.0004 | 187 |
| rs1801689 | 17 | 64210580 | C | A | 0.0832757 | 0.00557678 | -0.2858 | 0.3239 | 0.8536 | 0.4742 | 0.0005 | 223 |
| rs185263492 | 2 | 43996767 | A | T | 0.0242368 | 0.0026198 | 0.0251 | 0.0858 | 0.2827 | 0.1236 | 0.0002 | 86 |
| rs1883711 | 20 | 39179822 | C | G | 0.156174 | 0.0056001 | -0.0902 | 0.1332 | 0.0101 | 0.1928 | 0.0018 | 778 |
| rs189498338 | 1 | 109556113 | A | T | -0.0214633 | 0.00389357 | -0.3564 | 0.1485 | -0.2789 | 0.2136 | 0.0001 | 30 |
| rs1896995 | 10 | 65365385 | T | C | 0.0127252 | 0.00192851 | 0.0057 | 0.0649 | 0.0166 | 0.0937 | 0.0001 | 44 |
| rs1963676 | 21 | 40709960 | T | C | 0.0189758 | 0.00194707 | -0.0546 | 0.0684 | -0.1961 | 0.0991 | 0.0002 | 95 |
| rs203273 | 4 | 40418670 | T | C | 0.0139005 | 0.00201444 | 0.0095 | 0.0661 | -0.1154 | 0.0955 | 0.0001 | 48 |
| rs2066714 | 9 | 107586753 | C | T | 0.0292953 | 0.00285967 | 0.0487 | 0.111 | 0.0958 | 0.1601 | 0.0002 | 105 |
| rs2068888 | 10 | 94839642 | A | G | -0.0275689 | 0.00192443 | 0.0586 | 0.0645 | -0.007 | 0.0931 | 0.0005 | 205 |
| rs2073547 | 7 | 44582331 | G | A | 0.0488167 | 0.00245287 | 0.1316 | 0.069 | -0.0557 | 0.0996 | 0.0009 | 396 |
| rs2081048 | 19 | 33753166 | C | T | -0.0111302 | 0.00196305 | -0.0338 | 0.0686 | -0.0775 | 0.0989 | 0.0001 | 32 |
| rs2122982 | 12 | 57781893 | A | G | -0.0230697 | 0.00224176 | -0.0342 | 0.0757 | -0.1081 | 0.1094 | 0.0002 | 106 |
| rs213499 | 1 | 54866285 | C | A | -0.0112731 | 0.00189239 | 0.0219 | 0.0646 | 0.1845 | 0.0932 | 0.0001 | 35 |
| rs216140 | 5 | 149443298 | T | C | 0.0124318 | 0.00214428 | 0.0515 | 0.0814 | 0.0406 | 0.1171 | 0.0001 | 34 |
| rs2220729 | 12 | 89929953 | A | G | 0.0128201 | 0.00201258 | 0.0738 | 0.0678 | -0.0485 | 0.098 | 0.0001 | 41 |
| rs2238162 | 13 | 32959199 | T | C | -0.0251239 | 0.00192262 | -0.0091 | 0.065 | -0.0344 | 0.0939 | 0.0004 | 171 |
| rs224424 | 20 | 34147998 | G | A | -0.0260843 | 0.00235348 | -0.1003 | 0.0813 | -0.0588 | 0.1171 | 0.0003 | 123 |
| rs2250802 | 10 | 113921354 | A | G | -0.0252871 | 0.00214648 | 0.0598 | 0.0694 | -0.0679 | 0.1 | 0.0003 | 139 |
| rs2347699 | 7 | 134390964 | A | T | -0.0121069 | 0.00217157 | 0.2063 | 0.0783 | -0.0992 | 0.1134 | 0.0001 | 31 |
| rs2382818 | 2 | 219155907 | T | A | 0.0115756 | 0.00192906 | 0.0198 | 0.0693 | -0.0398 | 0.1003 | 0.0001 | 36 |
| rs2384008 | 2 | 24373150 | G | A | 0.0140236 | 0.0019676 | -0.0255 | 0.0705 | -0.1773 | 0.1017 | 0.0001 | 51 |
| rs2395617 | 6 | 35285720 | C | A | -0.0177128 | 0.0029086 | -0.115 | 0.1165 | 0.1576 | 0.1684 | 0.0001 | 37 |
| rs2413338 | 22 | 35663523 | T | C | -0.0123633 | 0.00200533 | 0.102 | 0.0656 | -0.001 | 0.0948 | 0.0001 | 38 |
| rs2439222 | 3 | 98613773 | T | C | -0.013561 | 0.00197958 | 0.1255 | 0.066 | -0.0817 | 0.0954 | 0.0001 | 47 |
| rs246179 | 16 | 14392641 | C | G | -0.011403 | 0.00197781 | 0.0747 | 0.066 | 0.0414 | 0.0954 | 0.0001 | 33 |
| rs2519093 | 9 | 136141870 | T | C | 0.079807 | 0.00247275 | -0.0463 | 0.0804 | -0.3457 | 0.1163 | 0.0024 | 1042 |
| rs2522062 | 5 | 131805416 | G | A | -0.0204169 | 0.00244858 | -0.1335 | 0.0784 | 0.0026 | 0.1127 | 0.0002 | 70 |
| rs2577611 | 2 | 109081066 | T | G | 0.0184959 | 0.00239908 | -0.0085 | 0.0739 | -0.0225 | 0.1064 | 0.0001 | 59 |
| rs2618567 | 20 | 17844492 | T | G | -0.036634 | 0.0020236 | -0.0498 | 0.0686 | 0.0801 | 0.099 | 0.0008 | 328 |
| rs262680 | 1 | 2167149 | T | C | -0.0130691 | 0.00191099 | 0.0749 | 0.0647 | -0.0186 | 0.0934 | 0.0001 | 47 |
| rs267733 | 1 | 150958836 | G | A | -0.0187326 | 0.00256622 | 0.0942 | 0.0909 | -0.1125 | 0.1316 | 0.0001 | 53 |
| rs2738447 | 19 | 11227480 | C | A | 0.0591662 | 0.00188722 | 0.0883 | 0.0659 | 0.0377 | 0.0952 | 0.0023 | 983 |
| rs2740488 | 9 | 107661742 | C | A | -0.0374266 | 0.00217187 | 0.1186 | 0.0807 | -0.036 | 0.1171 | 0.0007 | 297 |
| rs2773807 | 9 | 135993044 | G | A | -0.0117053 | 0.00192376 | -0.0463 | 0.0644 | 0.0052 | 0.0931 | 0.0001 | 37 |
| rs28406917 | 7 | 21449451 | T | C | 0.0152282 | 0.00193928 | 0.0437 | 0.0648 | 0.1397 | 0.0936 | 0.0001 | 62 |
| rs28471687 | 8 | 72459582 | G | A | 0.020205 | 0.00355787 | -0.0266 | 0.1709 | -0.076 | 0.2454 | 0.0001 | 32 |
| rs28615248 | 8 | 55451193 | C | T | 0.0300403 | 0.00241463 | -0.0756 | 0.0783 | 0.0377 | 0.1125 | 0.0004 | 155 |
| rs28807203 | 19 | 45173951 | C | A | -0.13655 | 0.00430913 | -0.0044 | 0.1318 | -0.2871 | 0.1897 | 0.0023 | 1004 |
| rs2911987 | 8 | 6564576 | G | A | 0.0136165 | 0.0020516 | 1.00E-04 | 0.0686 | 0.1162 | 0.0992 | 0.0001 | 44 |
| rs2972146 | 2 | 227100698 | T | G | 0.0112509 | 0.00197461 | 0.0301 | 0.067 | 0.0716 | 0.0966 | 0.0001 | 32 |
| rs308915 | 2 | 86859298 | C | A | -0.0119831 | 0.00194322 | 0.0979 | 0.0654 | -0.0143 | 0.0945 | 0.0001 | 38 |
| rs31226 | 5 | 53327571 | C | T | -0.0109438 | 0.00196562 | -0.0065 | 0.0658 | 0.0707 | 0.095 | 0.0001 | 31 |
| rs34023524 | 9 | 19327897 | C | T | 0.0350765 | 0.00356721 | -0.0438 | 0.1364 | 0.2659 | 0.1977 | 0.0002 | 97 |
| rs34042070 | 16 | 72101525 | G | C | 0.0700152 | 0.00246375 | -0.0948 | 0.0813 | 0.0125 | 0.1171 | 0.0019 | 808 |
| rs34815285 | 1 | 155094271 | C | T | 0.0125348 | 0.00189681 | -0.0296 | 0.0646 | 0.0458 | 0.0933 | 0.0001 | 44 |
| rs34914463 | 17 | 7366619 | C | T | -0.0171311 | 0.00283229 | -0.1781 | 0.1262 | -0.0128 | 0.1833 | 0.0001 | 37 |
| rs35081008 | 19 | 58662235 | T | C | -0.0322757 | 0.00262353 | 0.1336 | 0.0953 | 0.1907 | 0.1379 | 0.0004 | 151 |
| rs35243054 | 1 | 25821671 | T | G | -0.0292634 | 0.00189744 | 0.1058 | 0.0646 | -0.1645 | 0.0934 | 0.0006 | 238 |
| rs35570186 | 20 | 39288654 | A | G | 0.0299265 | 0.00510483 | -0.0496 | 0.1401 | 0.2032 | 0.2022 | 0.0001 | 34 |
| rs35814089 | 6 | 101433692 | C | T | 0.0112247 | 0.00191091 | -0.0988 | 0.0649 | -0.0633 | 0.0937 | 0.0001 | 35 |
| rs369298568 | 18 | 55319032 | C | T | 0.020139 | 0.00291926 | -0.2412 | 0.0998 | -0.1813 | 0.1439 | 0.0001 | 48 |
| rs3732359 | 3 | 119536429 | A | G | -0.0160503 | 0.00231512 | -0.0928 | 0.0695 | 0.0306 | 0.1003 | 0.0001 | 48 |
| rs3775228 | 4 | 87985166 | T | C | 0.0185041 | 0.00196215 | -0.041 | 0.066 | 0.0442 | 0.0952 | 0.0002 | 89 |
| rs3780181 | 9 | 2640759 | G | A | -0.0385591 | 0.00384155 | -0.1724 | 0.1421 | 0.157 | 0.2052 | 0.0002 | 101 |
| rs3820897 | 2 | 3642361 | C | T | -0.0189654 | 0.00248214 | 0.0335 | 0.0681 | -0.0744 | 0.0983 | 0.0001 | 58 |
| rs3823376 | 6 | 29944184 | T | C | 0.0176467 | 0.00190626 | -0.0241 | 0.0658 | -0.1106 | 0.0956 | 0.0002 | 86 |
| rs3824667 | 10 | 8100125 | G | A | 0.0141513 | 0.00255074 | 0.0235 | 0.0864 | 0.0792 | 0.1253 | 0.0001 | 31 |
| rs41280463 | 4 | 154191226 | A | G | -0.0196581 | 0.00257945 | 0.0029 | 0.0817 | -0.0303 | 0.1179 | 0.0001 | 58 |
| rs4148810 | 7 | 87102251 | T | C | -0.0209187 | 0.00248633 | 0.0286 | 0.0811 | -0.0453 | 0.1173 | 0.0002 | 71 |
| rs41785 | 7 | 116486020 | A | C | -0.0111724 | 0.00194196 | 0.0409 | 0.066 | -0.0248 | 0.0954 | 0.0001 | 33 |
| rs4299376 | 2 | 44072576 | T | G | -0.0779529 | 0.00202069 | -0.0024 | 0.0779 | -0.019 | 0.1128 | 0.0034 | 1488 |
| rs4307732 | 11 | 126244955 | A | G | 0.0573325 | 0.00311171 | -0.1014 | 0.0895 | 0.1562 | 0.129 | 0.0008 | 339 |
| rs4374942 | 7 | 155026807 | C | T | 0.0259845 | 0.00364859 | -0.1212 | 0.0899 | -0.0419 | 0.1294 | 0.0001 | 51 |
| rs4607926 | 1 | 171088413 | G | A | -0.0125245 | 0.002292 | 0.1179 | 0.0788 | -0.1106 | 0.1141 | 0.0001 | 30 |
| rs4616688 | 3 | 160042459 | T | G | -0.013898 | 0.00192044 | 0.0093 | 0.0658 | 0.0328 | 0.0951 | 0.0001 | 52 |
| rs463599 | 6 | 160545394 | T | G | 0.0531173 | 0.0027429 | 0.0291 | 0.0988 | 0.1555 | 0.1432 | 0.0009 | 375 |
| rs4689088 | 4 | 7222253 | A | G | 0.0146061 | 0.001972 | 0.0266 | 0.0667 | -0.0021 | 0.0963 | 0.0001 | 55 |
| rs472495 | 1 | 55521313 | T | G | 0.0546333 | 0.00197866 | -0.0706 | 0.0654 | -0.021 | 0.0943 | 0.0018 | 762 |
| rs4751996 | 10 | 118397894 | G | A | 0.0164643 | 0.00191883 | -0.0585 | 0.0668 | 0.1466 | 0.0966 | 0.0002 | 74 |
| rs542049 | 1 | 109696333 | C | T | -0.0207965 | 0.00201843 | 0.0293 | 0.0717 | -0.0141 | 0.1035 | 0.0002 | 106 |
| rs55637835 | 1 | 55466303 | T | C | -0.0239567 | 0.00294683 | 0.1617 | 0.1074 | -0.1754 | 0.1549 | 0.0002 | 66 |
| rs55691818 | 19 | 59056752 | G | C | 0.0171018 | 0.00238008 | -0.0319 | 0.0808 | 0.0407 | 0.1167 | 0.0001 | 52 |
| rs55714927 | 17 | 7080316 | T | C | -0.0397688 | 0.00243653 | 0.0947 | 0.0744 | -0.008 | 0.1071 | 0.0006 | 266 |
| rs557933 | 1 | 234853268 | C | A | 0.044949 | 0.00189236 | -0.0528 | 0.065 | -0.0088 | 0.0938 | 0.0013 | 564 |
| rs56000661 | 17 | 73878654 | C | A | -0.0207389 | 0.002068 | -0.0532 | 0.0765 | 0.1557 | 0.1105 | 0.0002 | 101 |
| rs56265089 | 16 | 72259360 | A | G | -0.0487402 | 0.00569811 | 0.0459 | 0.3373 | 0.3668 | 0.494 | 0.0002 | 73 |
| rs57159332 | 14 | 64233717 | C | T | 0.0223575 | 0.00281666 | 0.0445 | 0.0943 | -0.0327 | 0.1366 | 0.0001 | 63 |
| rs5754102 | 22 | 21927231 | A | C | -0.0141391 | 0.00252027 | 0.0474 | 0.0699 | -0.1254 | 0.1006 | 0.0001 | 31 |
| rs58148580 | 4 | 124758773 | T | C | 0.0198041 | 0.00305834 | 0.001 | 0.1019 | 0.0875 | 0.1476 | 0.0001 | 42 |
| rs58542926 | 19 | 19379549 | T | C | -0.132092 | 0.00352508 | 0.0511 | 0.1313 | 0.2608 | 0.1901 | 0.0032 | 1404 |
| rs59328596 | 8 | 21928227 | A | G | -0.0234829 | 0.00268649 | 0.0051 | 0.0846 | 0.096 | 0.1219 | 0.0002 | 76 |
| rs6022850 | 20 | 52535400 | T | C | 0.0125912 | 0.00194047 | 0.0965 | 0.065 | 0.095 | 0.0938 | 0.0001 | 42 |
| rs603424 | 10 | 102075479 | A | G | 0.021614 | 0.00254438 | -0.0261 | 0.1006 | 0.0904 | 0.1457 | 0.0002 | 72 |
| rs60847460 | 10 | 113983758 | T | C | -0.0159145 | 0.00274091 | -0.1174 | 0.0958 | -0.0933 | 0.139 | 0.0001 | 34 |
| rs60852193 | 3 | 147032401 | A | G | 0.0128346 | 0.00224731 | -0.1198 | 0.0916 | -0.0324 | 0.1325 | 0.0001 | 33 |
| rs6090040 | 20 | 62692060 | C | A | -0.0170833 | 0.00192902 | 0.1023 | 0.0649 | 0.1678 | 0.0937 | 0.0002 | 78 |
| rs6093446 | 20 | 39780932 | A | G | 0.0324432 | 0.00211743 | 0.0897 | 0.0748 | -0.0666 | 0.108 | 0.0005 | 235 |
| rs6139114 | 20 | 392335 | G | C | -0.0212079 | 0.00346407 | -0.1259 | 0.1242 | 0.0342 | 0.1795 | 0.0001 | 37 |
| rs61468422 | 11 | 66183248 | A | G | -0.0179139 | 0.00243724 | -7.00E-04 | 0.0841 | 0.0445 | 0.1213 | 0.0001 | 54 |
| rs61750929 | 9 | 91495135 | T | C | -0.0243212 | 0.00418258 | 0.1399 | 0.1762 | 0.0161 | 0.256 | 0.0001 | 34 |
| rs61871243 | 11 | 2998622 | A | G | 0.018856 | 0.00288791 | 0.1161 | 0.1078 | -0.1503 | 0.1555 | 0.0001 | 43 |
| rs62008532 | 15 | 62365877 | C | T | 0.0112369 | 0.00193792 | 0.1028 | 0.065 | -0.1015 | 0.0938 | 0.0001 | 34 |
| rs62072466 | 17 | 18115544 | A | T | 0.0178362 | 0.00243944 | 0.1902 | 0.092 | -0.1261 | 0.1325 | 0.0001 | 53 |
| rs62075819 | 17 | 46982544 | T | C | 0.0134751 | 0.00200233 | 0.0817 | 0.0675 | 0.1464 | 0.0974 | 0.0001 | 45 |
| rs62219001 | 21 | 16582880 | T | G | -0.0530653 | 0.00748012 | -0.1722 | 0.3844 | -1.0368 | 0.5554 | 0.0001 | 50 |
| rs62305783 | 4 | 100293200 | T | C | 0.0218533 | 0.00332953 | -0.0986 | 0.0929 | 0.0895 | 0.1339 | 0.0001 | 43 |
| rs62509311 | 8 | 74907295 | T | A | -0.0159506 | 0.00212802 | -0.0774 | 0.072 | 0.0131 | 0.1039 | 0.0001 | 56 |
| rs62544387 | 9 | 33163523 | G | C | -0.0148144 | 0.00228849 | 0.0352 | 0.0722 | 0.1307 | 0.1041 | 0.0001 | 42 |
| rs6602909 | 13 | 114551993 | C | T | 0.0296194 | 0.00205245 | 0.1373 | 0.0679 | -0.1751 | 0.0982 | 0.0005 | 208 |
| rs6606731 | 12 | 109982578 | A | T | 0.0159681 | 0.00242777 | 0.1031 | 0.0985 | -0.2167 | 0.1419 | 0.0001 | 43 |
| rs666334 | 2 | 45884213 | A | G | 0.0141091 | 0.00209008 | -0.0635 | 0.0789 | -0.0131 | 0.1139 | 0.0001 | 46 |
| rs6709904 | 2 | 44080324 | G | A | -0.0505577 | 0.00299811 | 0.0576 | 0.1065 | 0.1933 | 0.1533 | 0.0007 | 284 |
| rs67560206 | 5 | 75591986 | C | G | 0.0276835 | 0.00355832 | 0.0681 | 0.1024 | -0.0339 | 0.1481 | 0.0001 | 61 |
| rs6785233 | 3 | 170756985 | G | T | 0.0277941 | 0.00353741 | -0.0166 | 0.167 | 1.00E-04 | 0.2435 | 0.0001 | 62 |
| rs68023264 | 17 | 27686313 | A | G | -0.021396 | 0.00273892 | -0.0206 | 0.0847 | -0.0788 | 0.1221 | 0.0001 | 61 |
| rs6882076 | 5 | 156390297 | C | T | 0.0455741 | 0.00198294 | 0.0724 | 0.0685 | -0.1307 | 0.099 | 0.0012 | 528 |
| rs6896005 | 5 | 122834112 | C | T | 0.0199662 | 0.00192092 | 0.0617 | 0.0646 | -0.1059 | 0.0931 | 0.0003 | 108 |
| rs7005453 | 8 | 126629700 | A | G | 0.0135228 | 0.00235313 | 0.0214 | 0.0802 | 0.0706 | 0.1159 | 0.0001 | 33 |
| rs704 | 17 | 26694861 | A | G | 0.0192243 | 0.00191489 | 0.11 | 0.0652 | -0.1368 | 0.0941 | 0.0002 | 101 |
| rs7108486 | 11 | 5677158 | C | T | -0.0439372 | 0.00634523 | 0.0832 | 0.219 | -0.2308 | 0.3169 | 0.0001 | 48 |
| rs71311871 | 3 | 58420613 | G | A | -0.0400404 | 0.00347381 | -0.0608 | 0.1204 | -0.0542 | 0.1728 | 0.0003 | 133 |
| rs7187512 | 16 | 56840171 | G | A | -0.0109739 | 0.00193603 | 0.0086 | 0.0675 | -0.0655 | 0.0976 | 0.0001 | 32 |
| rs7204 | 5 | 139624864 | C | T | -0.0160365 | 0.00234828 | 0.0277 | 0.0723 | -0.1508 | 0.1044 | 0.0001 | 47 |
| rs72631343 | 17 | 67191270 | G | C | -0.04404 | 0.00285567 | -0.0466 | 0.0933 | -0.2314 | 0.1337 | 0.0006 | 238 |
| rs72784625 | 2 | 8720650 | G | T | -0.0125258 | 0.00215358 | 0.0533 | 0.0694 | 0.0398 | 0.1 | 0.0001 | 34 |
| rs72848251 | 6 | 32602483 | A | G | 0.0460899 | 0.00246858 | 0.0123 | 0.1022 | -0.021 | 0.1481 | 0.0008 | 349 |
| rs7300192 | 12 | 51794043 | G | A | -0.0174791 | 0.00198948 | 0.0487 | 0.0684 | -0.0512 | 0.0988 | 0.0002 | 77 |
| rs73015024 | 19 | 11197598 | T | G | -0.219509 | 0.00286668 | -0.0231 | 0.1072 | -0.2554 | 0.1548 | 0.0134 | 5863 |
| rs73075609 | 20 | 5580789 | T | C | 0.0490853 | 0.0059639 | -0.7199 | 0.3267 | -0.1064 | 0.4769 | 0.0002 | 68 |
| rs7316368 | 12 | 617210 | C | T | 0.0157077 | 0.00216882 | -0.0855 | 0.0835 | 0.0241 | 0.1206 | 0.0001 | 52 |
| rs739431 | 3 | 50255305 | G | A | -0.0152048 | 0.00277482 | -0.0128 | 0.0912 | -0.1587 | 0.1317 | 0.0001 | 30 |
| rs74035509 | 16 | 88567333 | T | C | 0.0265066 | 0.00358126 | 0.1642 | 0.1439 | -0.1446 | 0.2086 | 0.0001 | 55 |
| rs74341202 | 3 | 132183991 | A | G | -0.0476176 | 0.00420924 | 0.1905 | 0.1349 | 0.1488 | 0.1955 | 0.0003 | 128 |
| rs7516453 | 1 | 15972558 | G | C | -0.0123612 | 0.00212883 | 0.027 | 0.0684 | -0.0859 | 0.0988 | 0.0001 | 34 |
| rs7551451 | 1 | 235110095 | G | A | -0.0216138 | 0.00236124 | 0.1506 | 0.0898 | -0.0684 | 0.1302 | 0.0002 | 84 |
| rs75588192 | 12 | 133048600 | A | G | 0.0197578 | 0.00282447 | 0.0039 | 0.0975 | -0.1687 | 0.1405 | 0.0001 | 49 |
| rs7562734 | 2 | 63062671 | C | G | -0.0260008 | 0.00202792 | 0.0764 | 0.0659 | 0.0329 | 0.0952 | 0.0004 | 164 |
| rs7569317 | 2 | 203527979 | C | T | 0.0254243 | 0.00189143 | 0.1331 | 0.0648 | 0.1555 | 0.0934 | 0.0004 | 181 |
| rs75735512 | 15 | 57047196 | G | A | 0.0333884 | 0.00466944 | -0.0494 | 0.187 | 0.5358 | 0.27 | 0.0001 | 51 |
| rs75816352 | 13 | 111038803 | T | C | 0.0151199 | 0.00201702 | 0.0398 | 0.0659 | -0.0606 | 0.0951 | 0.0001 | 56 |
| rs7603427 | 2 | 204317553 | T | C | 0.0138321 | 0.00189786 | -0.0018 | 0.0651 | -0.0553 | 0.0939 | 0.0001 | 53 |
| rs76147254 | 1 | 28574497 | C | G | -0.0204889 | 0.00375412 | -0.0969 | 0.1508 | 0.1427 | 0.2178 | 0.0001 | 30 |
| rs76775468 | 8 | 28843630 | C | T | 0.0286419 | 0.00485427 | 0.1685 | 0.1899 | 0.2267 | 0.2753 | 0.0001 | 35 |
| rs76895963 | 12 | 4384844 | G | T | -0.0709513 | 0.00741143 | -0.3442 | 0.1954 | -0.2419 | 0.2817 | 0.0002 | 92 |
| rs7695536 | 4 | 57955234 | C | T | -0.011118 | 0.00193366 | -0.0184 | 0.0649 | -0.0038 | 0.0937 | 0.0001 | 33 |
| rs76984261 | 19 | 45635036 | T | A | 0.0408799 | 0.00413894 | -0.06 | 0.1492 | 0.2815 | 0.2164 | 0.0002 | 98 |
| rs7746081 | 6 | 16126934 | A | G | -0.0334797 | 0.00207864 | -0.0203 | 0.0669 | 0.1405 | 0.0966 | 0.0006 | 259 |
| rs77542162 | 17 | 67081278 | G | A | 0.17887 | 0.00645012 | 0.337 | 0.3808 | -0.0751 | 0.5654 | 0.0018 | 769 |
| rs77960347 | 18 | 47109955 | G | A | 0.0851286 | 0.00836788 | -0.8817 | 0.3738 | 0.1761 | 0.545 | 0.0002 | 103 |
| rs78058190 | 2 | 219699999 | A | G | 0.0270032 | 0.00486519 | -0.1368 | 0.1186 | 0.2655 | 0.1716 | 0.0001 | 31 |
| rs78173576 | 17 | 7217099 | G | T | -0.0341094 | 0.00510931 | 0.1497 | 0.1753 | -0.0786 | 0.2542 | 0.0001 | 45 |
| rs784882 | 12 | 53803633 | G | A | 0.0184095 | 0.00251897 | 0.0369 | 0.0871 | -0.0114 | 0.1256 | 0.0001 | 53 |
| rs7908745 | 10 | 45953767 | G | A | 0.0134821 | 0.00206234 | -0.0264 | 0.0671 | 0.1657 | 0.097 | 0.0001 | 43 |
| rs7960935 | 12 | 100789852 | C | T | -0.0144037 | 0.00193922 | 0.0111 | 0.0647 | 0.1526 | 0.0934 | 0.0001 | 55 |
| rs799157 | 7 | 73020301 | C | T | -0.0468863 | 0.00468473 | 0.5612 | 0.2124 | 0.1984 | 0.3054 | 0.0002 | 100 |
| rs79953563 | 11 | 32159750 | A | T | 0.0191638 | 0.00268353 | 7.00E-04 | 0.0827 | -0.0566 | 0.1194 | 0.0001 | 51 |
| rs8005362 | 14 | 70770867 | G | A | 0.0201149 | 0.00192716 | 0.0056 | 0.0645 | -0.0628 | 0.0932 | 0.0003 | 109 |
| rs80276949 | 10 | 52373245 | A | G | 0.0468997 | 0.00644767 | -0.2878 | 0.2847 | -0.3758 | 0.4132 | 0.0001 | 53 |
| rs8030799 | 15 | 91109374 | C | G | -0.0160446 | 0.0026849 | 0.0287 | 0.0842 | -0.0564 | 0.122 | 0.0001 | 36 |
| rs8103315 | 19 | 45254168 | A | C | 0.0630036 | 0.00279272 | -0.0258 | 0.0839 | -0.0256 | 0.1208 | 0.0012 | 509 |
| rs821840 | 16 | 56994528 | G | A | -0.0437432 | 0.00204737 | -0.0616 | 0.0718 | 0.1275 | 0.1037 | 0.0011 | 456 |
| rs867772 | 1 | 220972343 | G | A | 0.0311833 | 0.0020318 | -0.0181 | 0.0709 | -0.0966 | 0.1025 | 0.0005 | 236 |
| rs9287908 | 2 | 169828867 | T | C | -0.0251497 | 0.00193692 | 0.0252 | 0.0642 | 0.0883 | 0.0927 | 0.0004 | 169 |
| rs9297994 | 8 | 59392324 | A | G | -0.0411393 | 0.0020233 | 0.0485 | 0.0666 | 0.0606 | 0.0962 | 0.0010 | 413 |
| rs934197 | 2 | 21267461 | A | G | 0.112288 | 0.00200093 | 0.0386 | 0.0712 | 0.0774 | 0.103 | 0.0073 | 3149 |
| rs9389268 | 6 | 135419631 | G | A | -0.0276924 | 0.00218455 | 0.0385 | 0.0683 | 0.0339 | 0.0984 | 0.0004 | 161 |
| rs9403030 | 6 | 139347894 | C | T | -0.0109878 | 0.00194255 | -0.0857 | 0.0656 | -0.0294 | 0.0949 | 0.0001 | 32 |
| rs9471975 | 6 | 42919222 | C | T | -0.0146922 | 0.00193549 | 0.0678 | 0.065 | 0.0083 | 0.0938 | 0.0001 | 58 |
| rs9604529 | 13 | 114622597 | G | A | -0.020138 | 0.00244294 | 0.0184 | 0.0988 | -0.2038 | 0.1438 | 0.0002 | 68 |
| rs9616822 | 22 | 50840573 | A | G | 0.0158614 | 0.00201459 | 0.0327 | 0.0648 | -0.0244 | 0.0938 | 0.0001 | 62 |
| rs964184 | 11 | 116648917 | C | G | -0.106826 | 0.00280881 | -0.1001 | 0.091 | -0.0854 | 0.1313 | 0.0033 | 1446 |
| rs9673065 | 15 | 75180892 | G | T | 0.0130176 | 0.00194665 | 0.0471 | 0.0649 | 0.0432 | 0.0937 | 0.0001 | 45 |
| rs969075 | 20 | 17792323 | C | T | 0.0132764 | 0.00204072 | 0.0061 | 0.072 | 0.1065 | 0.1039 | 0.0001 | 42 |
| rs9834932 | 3 | 32535382 | G | A | -0.0379173 | 0.00335939 | 0.0041 | 0.1181 | 0.2706 | 0.1705 | 0.0003 | 127 |
| rs9871402 | 3 | 142024351 | C | A | -0.0116615 | 0.00193675 | 2.00E-04 | 0.0648 | 0.1414 | 0.0936 | 0.0001 | 36 |
| rs9894946 | 17 | 7571080 | G | A | -0.0235826 | 0.0026656 | -0.0066 | 0.0903 | -0.1426 | 0.1304 | 0.0002 | 78 |
| rs9987289 | 8 | 9183358 | G | A | 0.0567992 | 0.00331823 | 0.26 | 0.0972 | 0.255 | 0.14 | 0.0007 | 293 |

**Table S4. The genetic instruments of Total cholesterol used in Mendelian analysis.**

| **SNP** | **Chr** | **Pos** | **Effect_allele** | **Other_allele** | **Beta** | **Se** | **Beta.BE** | **Se.BE** | **Beta.EC** | **Se.EC** | **R^2^** | **F** |
| --- | --- | --- | --- | --- | --- | --- | --- | --- | --- | --- | --- | --- |
| rs102275 | 11 | 61557803 | C | T | -0.0660426 | 0.00418036 | 0.1077 | 0.0654 | 0.1094 | 0.0944 | 0.0022 | 250 |
| rs1057558 | 7 | 1062366 | C | T | -0.0280556 | 0.00482698 | -0.0498 | 0.0732 | -0.1027 | 0.1057 | 0.0003 | 34 |
| rs1081105 | 19 | 45412955 | C | A | 0.136691 | 0.0122076 | 0.0534 | 0.2237 | 0.1298 | 0.3249 | 0.0011 | 125 |
| rs11127048 | 2 | 27752463 | A | G | -0.0353702 | 0.00417022 | -0.1233 | 0.067 | 0.0301 | 0.0967 | 0.0006 | 72 |
| rs111278137 | 19 | 45215081 | A | G | -0.113707 | 0.014207 | -0.0927 | 0.3225 | 0.6611 | 0.462 | 0.0006 | 64 |
| rs11206517 | 1 | 55526428 | G | T | 0.0851169 | 0.0111899 | -0.0369 | 0.1541 | 0.0926 | 0.2229 | 0.0005 | 58 |
| rs115478735 | 9 | 136149711 | T | A | 0.0562229 | 0.00517162 | -0.053 | 0.0809 | -0.3533 | 0.1171 | 0.0010 | 118 |
| rs11591147 | 1 | 55505647 | T | G | -0.273043 | 0.0152309 | -0.0927 | 0.1772 | 0.1499 | 0.2543 | 0.0028 | 321 |
| rs11621792 | 14 | 24871926 | T | C | 0.0239578 | 0.0040379 | 0.0514 | 0.0667 | -0.0272 | 0.0964 | 0.0003 | 35 |
| rs1169292 | 12 | 121426478 | T | C | 0.0278408 | 0.00433611 | 0.0038 | 0.0678 | 0.0566 | 0.0977 | 0.0004 | 41 |
| rs11789603 | 9 | 107647019 | T | C | 0.0418447 | 0.00643476 | -0.0761 | 0.1206 | 0.1598 | 0.1753 | 0.0004 | 42 |
| rs12151108 | 19 | 11197261 | A | G | -0.132111 | 0.00614387 | -0.0223 | 0.1072 | -0.2555 | 0.1548 | 0.0040 | 462 |
| rs12740374 | 1 | 109817590 | T | G | -0.0929409 | 0.00480921 | -0.1444 | 0.0786 | 0.1275 | 0.1137 | 0.0032 | 373 |
| rs1461729 | 8 | 9187242 | G | A | 0.0754733 | 0.00664826 | 0.2555 | 0.0942 | 0.2263 | 0.1358 | 0.0011 | 129 |
| rs1500188 | 5 | 122827953 | G | A | -0.0242707 | 0.00402644 | -0.0654 | 0.0646 | 0.1287 | 0.0931 | 0.0003 | 36 |
| rs17699030 | 19 | 11330942 | G | A | -0.0651514 | 0.0110409 | 0.0616 | 0.15 | -0.059 | 0.2181 | 0.0003 | 35 |
| rs1800961 | 20 | 43042364 | T | C | -0.0805672 | 0.0116769 | -0.0777 | 0.1543 | 0.144 | 0.2227 | 0.0004 | 48 |
| rs1864163 | 16 | 56997233 | A | G | -0.0465851 | 0.0045502 | 0.115 | 0.0792 | -0.0871 | 0.1146 | 0.0009 | 105 |
| rs1883711 | 20 | 39179822 | C | G | 0.0840282 | 0.0117472 | -0.0902 | 0.1332 | 0.0101 | 0.1928 | 0.0004 | 51 |
| rs2326077 | 8 | 59385919 | T | C | -0.0224173 | 0.00423777 | 0.0535 | 0.0663 | 0.0657 | 0.0957 | 0.0002 | 28 |
| rs2495477 | 1 | 55518467 | G | A | -0.029019 | 0.00417434 | 0.0183 | 0.065 | -0.0264 | 0.0938 | 0.0004 | 48 |
| rs261290 | 15 | 58678720 | C | T | -0.078138 | 0.00421371 | -0.0993 | 0.0664 | -0.048 | 0.0958 | 0.0030 | 344 |
| rs2618566 | 20 | 17844684 | T | G | -0.0219844 | 0.0042221 | -0.0316 | 0.0693 | 0.0945 | 0.1002 | 0.0002 | 27 |
| rs2642438 | 1 | 220970028 | G | A | 0.0318207 | 0.00437814 | -0.011 | 0.0715 | -0.1558 | 0.1033 | 0.0005 | 53 |
| rs2740488 | 9 | 107661742 | C | A | -0.0425254 | 0.00453947 | 0.1186 | 0.0807 | -0.036 | 0.1171 | 0.0008 | 88 |
| rs2792735 | 10 | 113921825 | A | G | -0.0320407 | 0.00445386 | 0.0651 | 0.0686 | -0.0624 | 0.0988 | 0.0004 | 52 |
| rs28807203 | 19 | 45173951 | C | A | -0.10157 | 0.00926956 | -0.0044 | 0.1318 | -0.2871 | 0.1897 | 0.0010 | 120 |
| rs2943650 | 2 | 227105921 | T | C | -0.0220515 | 0.00417007 | 0.0273 | 0.067 | 0.0611 | 0.0966 | 0.0002 | 28 |
| rs34042070 | 16 | 72101525 | G | C | 0.0352287 | 0.00514834 | -0.0948 | 0.0813 | 0.0125 | 0.1171 | 0.0004 | 47 |
| rs35135293 | 2 | 20363666 | T | C | -0.0246633 | 0.00401709 | -0.0418 | 0.0654 | 0.2576 | 0.0949 | 0.0003 | 38 |
| rs35237252 | 8 | 19870271 | A | C | 0.0323113 | 0.00465699 | 0.0288 | 0.0764 | 0.0993 | 0.1104 | 0.0004 | 48 |
| rs3777411 | 6 | 160476945 | T | C | -0.0405292 | 0.00558926 | 0.1786 | 0.0961 | -0.0117 | 0.1391 | 0.0005 | 53 |
| rs4299376 | 2 | 44072576 | T | G | -0.0428972 | 0.00428182 | -0.0024 | 0.0779 | -0.019 | 0.1128 | 0.0009 | 100 |
| rs4307732 | 11 | 126244955 | A | G | 0.0364447 | 0.00651417 | -0.1014 | 0.0895 | 0.1562 | 0.129 | 0.0003 | 31 |
| rs4860948 | 4 | 69340991 | A | T | 0.0255436 | 0.00466595 | -0.0993 | 0.0757 | -0.177 | 0.1093 | 0.0003 | 30 |
| rs525028 | 11 | 116705516 | A | G | -0.035615 | 0.00441909 | 0.0267 | 0.0671 | 0.1335 | 0.097 | 0.0006 | 65 |
| rs553427 | 1 | 234852760 | T | C | 0.0312099 | 0.00399797 | -0.0562 | 0.065 | 0.0019 | 0.0938 | 0.0005 | 61 |
| rs562338 | 2 | 21288321 | G | A | 0.098242 | 0.00519045 | 0.0551 | 0.0827 | 0.0985 | 0.1193 | 0.0031 | 358 |
| rs56325564 | 17 | 45766771 | A | G | 0.0289563 | 0.00402858 | 0.0908 | 0.0647 | 0.1173 | 0.0934 | 0.0004 | 52 |
| rs58542926 | 19 | 19379549 | T | C | -0.0982855 | 0.00760933 | 0.0511 | 0.1313 | 0.2608 | 0.1901 | 0.0014 | 167 |
| rs633695 | 15 | 58725839 | G | A | 0.0587261 | 0.00441179 | 0.0786 | 0.07 | -0.0274 | 0.1012 | 0.0015 | 177 |
| rs653178 | 12 | 112007756 | T | C | 0.0305465 | 0.00400532 | 0.0713 | 0.0653 | 0.0914 | 0.0943 | 0.0005 | 58 |
| rs6882345 | 5 | 156397673 | A | G | 0.0359238 | 0.00414212 | 0.0711 | 0.0686 | -0.1311 | 0.099 | 0.0007 | 75 |
| rs72631343 | 17 | 67191270 | G | C | -0.0346131 | 0.0059663 | -0.0466 | 0.0933 | -0.2314 | 0.1337 | 0.0003 | 34 |
| rs72836561 | 17 | 41926126 | T | C | -0.0763491 | 0.0114495 | -0.3352 | 0.1829 | 0.2924 | 0.266 | 0.0004 | 44 |
| rs73013176 | 19 | 11147526 | C | T | -0.127568 | 0.0192153 | -0.3513 | 0.481 | -0.1323 | 0.6997 | 0.0004 | 44 |
| rs77542162 | 17 | 67081278 | G | A | 0.118862 | 0.0134466 | 0.337 | 0.3808 | -0.0751 | 0.5654 | 0.0007 | 78 |
| rs77960347 | 18 | 47109955 | G | A | 0.190079 | 0.0174651 | -0.8817 | 0.3738 | 0.1761 | 0.545 | 0.0010 | 118 |
| rs79220007 | 6 | 26098474 | C | T | -0.050516 | 0.00754059 | 0.1494 | 0.1695 | 0.0638 | 0.2473 | 0.0004 | 45 |
| rs8103315 | 19 | 45254168 | A | C | 0.0455886 | 0.00599703 | -0.0258 | 0.0839 | -0.0256 | 0.1208 | 0.0005 | 58 |
| rs9304381 | 18 | 47158234 | T | C | 0.0483354 | 0.00519129 | 0.1408 | 0.0842 | 0.1548 | 0.1219 | 0.0008 | 87 |

**Table S5. Causal effects of circulating lipids on Barrett’s Esophagus outcomes after excluding instrumental variables associated with confounding factors.**

| **Exposure** | **Outcome** | **Method** | **β(SE)** | **OR (95% CI)** | **Instrumental**  **variables (n)** | **P** |
| --- | --- | --- | --- | --- | --- | --- |
| Triglycerides | Barret Esophagus | IVW | 0.580(0.221) | 1.786(1.158-2.755) | 47 | 0.009 |
|  | Barret Esophagus | WM | 0.618(0.315) | 1.855(1.000-3.440) | 47 | 0.052 |
|  | Barret Esophagus | MR-Egger | 0.382(0.354) | 1.466(0.732-2.935) | 47 | 0.286 |
| HDL cholesterol | Barret Esophagus | IVW | 0.100(0.168) | 1.105(0.796-1.536) | 74 | 0.550 |
|  | Barret Esophagus | WM | -0.202(0.270) | 0.817(0.481-1.387) | 74 | 0.454 |
|  | Barret Esophagus | MR-Egger | 0.141(0.273) | 1.152(0.674-1.968) | 74 | 0.607 |
| LDL cholesterol | Barret Esophagus | IVW | 0.164(0.142) | 1.178(0.891-1.556) | 284 | 0.250 |
|  | Barret Esophagus | WM | 0.202(0.238) | 1.224(0.768-1.952) | 284 | 0.395 |
|  | Barret Esophagus | MR-Egger | 0.394(0.200) | 1.482(1.001-2.194) | 284 | 0.050 |
| Total cholesterol | Barret Esophagus | IVW | 0.271(0.239) | 1.312(0.821-2.095) | 51 | 0.256 |
|  | Barret Esophagus | WM | 0.338(0.367) | 1.402(0.683-2.880) | 51 | 0.357 |
|  | Barret Esophagus | MR-Egger | 0.445(0.446) | 1.561(0.651-3.742) | 51 | 0.323 |

**Table S6. Causal effects of circulating lipids on Esophageal Cancer outcomes after excluding instrumental variables associated with confounding factors.**

| **Exposure** | **Outcome** | **Method** | **β(SE)** | **OR (95% CI)** | **Instrumental**  **variables (n)** | **P** |
| --- | --- | --- | --- | --- | --- | --- |
| Triglycerides | esophageal cancer | IVW | 0.019(0.319) | 1.020(0.546-1.907) | 47 | 0.950 |
|  | esophageal cancer | WM | -0.140(0.471) | 0.869(0.345-2.187) | 47 | 0.766 |
|  | esophageal cancer | MR-Egger | -0.578(0.511) | 0.561(0.206-1.528) | 47 | 0.264 |
| HDL cholesterol | esophageal cancer | IVW | 0.237(0.249) | 1.268(0.778-2.066) | 74 | 0.341 |
|  | esophageal cancer | WM | 0.529(0.374) | 1.698(0.816-3.532) | 74 | 0.157 |
|  | esophageal cancer | MR-Egger | 0.648(0.401) | 1.912(0.870-4.200) | 74 | 0.111 |
| LDL cholesterol | esophageal cancer | IVW | -0.019(0.200) | 0.981(0.662-1.452) | 284 | 0.922 |
|  | esophageal cancer | WM | -0.0218(0.359) | 0.804(0.398-1.627) | 284 | 0.545 |
|  | esophageal cancer | MR-Egger | -0.099(0.283) | 0.905(0.520-1.575) | 284 | 0.724 |
| Total cholesterol | esophageal cancer | IVW | -0.080(0.350) | 0.923(0.465-1.834) | 51 | 0.819 |
|  | esophageal cancer | WM | 0.251(0.506) | 1.285(0.477-3.466) | 51 | 0.620 |
|  | esophageal cancer | MR-Egger | 0.475(0.648) | 1.609(0.452-5.728) | 51 | 0.467 |

**Table S7. The outcomes of sensitivity MR analyses of circulating lipids on esophageal cancer.**

| **exposure** | **MR-PRESSO** | **IVW Estimates** | | **R-Egger Pleiotropy Test** | |
| --- | --- | --- | --- | --- | --- |
|  | **Global p-value** | **Cochran’s Q** | **P-value** | **MR-Egger Intercept** | **p-Value** |
| Triglycerides | 0.953 | 31.600 | 0.948 | 0.037 | 0.141 |
| HDL cholesterol | 0.339 | 78.760 | 0.302 | -0.028 | 0.197 |
| LDL cholesterol | 0.708 | 268.999 | 0.716 | 0.004 | 0.687 |
| Total cholesterol | 0.158 | 50 | 0.168 | -0.035 | 0.313 |

**Table S8. Causal effects of Barrett’s Esophagus on circulating lipids outcomes**

| **outcomes** | **Method** | **No. of SNPs** | **OR (95% CI)** | **P for association** | **P for heterogeneity test** | **P for MR-Egger intercept** |
| --- | --- | --- | --- | --- | --- | --- |
| Triglycerides | IVW | 4 | 1.001(0.993-1.021) | 0.327 | 0.533 | 0.379 |
|  | WM | 4 | 1.000(0.983-1.018) | 0.980 | - | - |
|  | MR-Egger | 4 | 0.807(0.548-1.189) | 0.392 | 0.626 | - |
| HDL cholesterol | IVW | 4 | 0.993(0.977-1.009) | 0.365 | 0.287 | 0.210 |
|  | WM | 4 | 0.993(0.975-1.011) | 0.460 | - | - |
|  | MR-Egger | 4 | 1.438(0.965-2.141) | 0.216 | 0.801 | - |
| LDL cholesterol | IVW | 16 | 0.999(0.996-1.002) | 0.543 | 0.030 | 0.601 |
|  | WM | 16 | 0.999(0.996-1.004) | 0.887 | - | - |
|  | MR-Egger | 16 | 0.998(0.993-1.003) | 0.439 | 0.023 | - |
| Total cholesterol | IVW | 16 | 1.001(0.996-1.006) | 0.755 | 0.585 | 0.354 |
|  | WM | 16 | 1.000(0.993-1.007) | 0.973 | - | - |
|  | MR-Egger | 16 | 0.998(0.991-1.005) | 0.635 | 0.581 | - |

**Table S9 Causal effects of Esophageal Cancer on circulating lipids outcomes**

| **outcomes** | **Method** | **No. of SNPs** | **OR (95% CI)** | **P for association** | **P for heterogeneity test** | **P for MR-Egger intercept** |
| --- | --- | --- | --- | --- | --- | --- |
| Triglycerides | IVW | 4 | 1.007(0.991-1.024) | 0.394 | 0.093 | 0.337 |
|  | WM | 4 | 0.999(0.984-1.015) | 0.939 | - | - |
|  | MR-Egger | 4 | 1.058(0.978-1.145) | 0.294 | 0.165 | - |
| HDL cholesterol | IVW | 4 | 1.002(0.981-1.022) | 0.871 | 0.028 | 0.913 |
|  | WM | 4 | 0.994(0.976-1.012) | 0.511 | - | - |
|  | MR-Egger | 4 | 1.010(0.889-1.147) | 0.896 | 0.011 | - |
| LDL cholesterol | IVW | 21 | 0.998(0.995-1.001) | 0.252 | 5.726E-15 | 0.082 |
|  | WM | 21 | 0.999(0.997-1.001) | 0.516 | - | - |
|  | MR-Egger | 21 | 1.002(0.997-1.006) | 0.537 | 2.871E-12 | - |
| Total cholesterol | IVW | 21 | 0.998(0.995-1.002) | 0.427 | 0.027 | 0.154 |
|  | WM | 21 | 0.997(0.993-1.002) | 0.224 | - | - |
|  | MR-Egger | 21 | 1.002(0.996-1.007) | 0.549 | 0.047 | - |

**Table S10. MVMR analysis between circulating lipids and Barrett’s Esophagus outcomes**

| **Exposure** | **β(SE)** | **OR (95% CI)** | **Instrumental**  **variables (n)** | **P** |
| --- | --- | --- | --- | --- |
| Triglycerides | 0.583(0.284) | 1.791(1.027-3.122) | 22 | 0.041 |
| HDL cholesterol | 0.562(0.336) | 1.754(0.908-3.389) | 35 | 0.094 |
| LDL cholesterol | 0.701(0.476) | 2.016(0.793-5.124) | 122 | 0.141 |
| Total cholesterol | -0.661(0.699) | 0.517(0.131-2.034) | 32 | 0.344 |
| Gastroesophageal reflux | 0.446(0.459) | 1.562(0.635-3.841) | 20 | 0.331 |
| Body mass index | 0.030(0.471) | 1.030(0.409-2.594) | 151 | 0.950 |

**Table S11. MVMR analysis between circulating lipids and Barrett’s Esophagus outcomes**

| **Exposure** | **β(SE)** | **OR (95% CI)** | **Instrumental**  **variables (n)** | **P** |
| --- | --- | --- | --- | --- |
| Triglycerides | 0.558(0.267) | 1.747(1.035-2.949) | 23 | 0.037 |
| HDL cholesterol | 0.070(0.304) | 1.073(0.591-1.946) | 42 | 0.817 |
| LDL cholesterol | -0.022(0.442) | 0.978(0.411-2.326) | 178 | 0.960 |
| Total cholesterol | 0.389(0.670) | 1.476(0.397-5.486) | 36 | 0.562 |

**Table S12. MVMR analysis between circulating lipids and Esophageal Cancer outcomes after adjusting for GR and BMI.**

| **Exposure** | **β(SE)** | **OR (95% CI)** | **Instrumental**  **variables (n)** | **P** |
| --- | --- | --- | --- | --- |
| Triglycerides | -0.243(0.411) | 0.784(0.350-1.755) | 22 | 0.556 |
| HDL cholesterol | 0.537(0.487) | 1.711(0.659-4.444) | 36 | 0.270 |
| LDL cholesterol | 0.492(0.691) | 1.636(0.422-6.337) | 122 | 0.476 |
| Total cholesterol | -0.813(1.014) | 0.444(0.061-3.236) | 32 | 0.423 |
| Gastroesophageal reflux | 0.010(0.664) | 1.010(0.275-3.712) | 20 | 0.988 |
| Body mass index | 0.454(0.682) | 1.575(0.414-5.994) | 151 | 0.505 |

**Table S13. MVMR analysis between circulating lipids and Esophageal Cancer outcomes.**

| **Exposure** | **β(SE)** | **OR (95% CI)** | **Instrumental**  **variables (n)** | **P** |
| --- | --- | --- | --- | --- |
| **Triglycerides** | **-0.283(0.377)** | **0.754(0.360-1.578)** | **23** | **0.454** |
| **HDL cholesterol** | **0.659(0.428)** | **1.933(0.835-4.472)** | **42** | **0.124** |
| **LDL cholesterol** | **0.809(0.624)** | **2.246(0.661-7.630)** | **178** | **0.195** |
| **Total cholesterol** | **-1.232(0.944)** | **0.292(0.046-1.856)** | **36** | **0.192** |

**Fig S1. Scatter plots for MR analyses of the causal effect of circulating lipids on Barrett's Esophagus.** A, triglyceride; B, HDL cholesterol; C, LDL cholesterol; D, Total cholesterol.


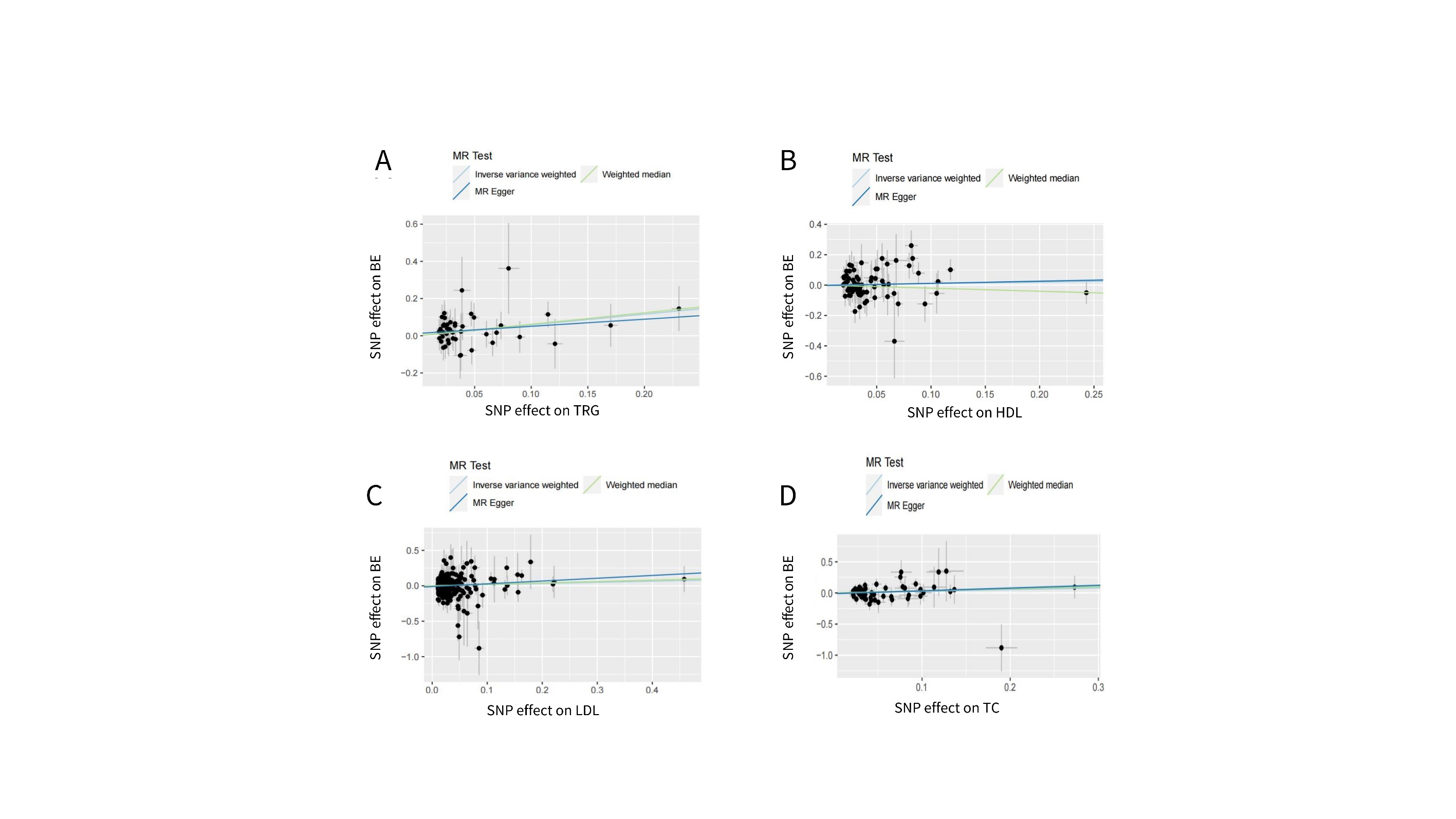


**Fig S2. Scatter plots for MR analyses of the causal effect of circulating lipids on Esophageal Cancer.** A, triglyceride; B, HDL cholesterol; C, LDL cholesterol; D, Total cholesterol.


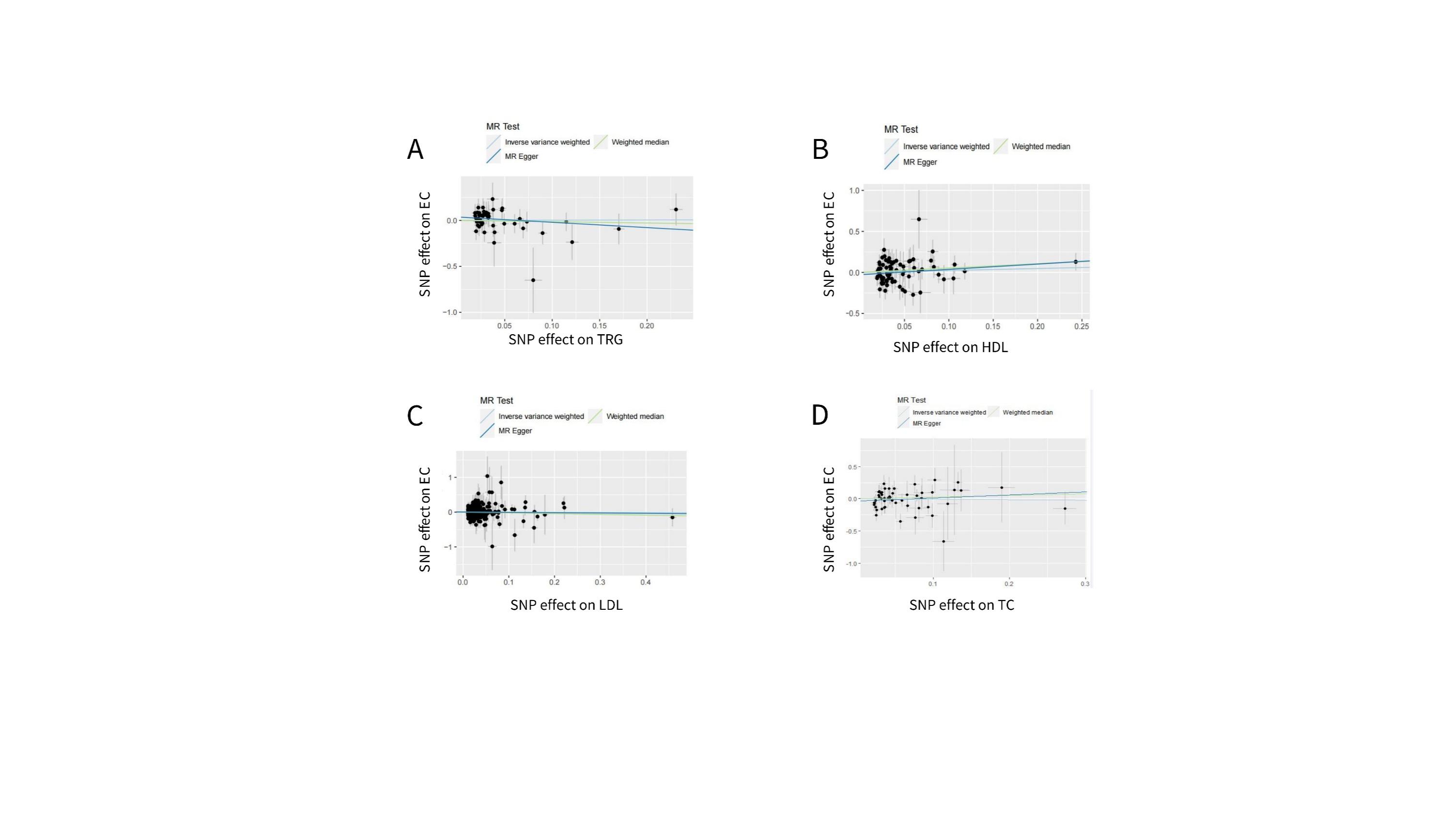


**Fig S3. The forest plot of leave-one-out sensitivity analysis for triglycerides on Barrett’s Esophagus.**


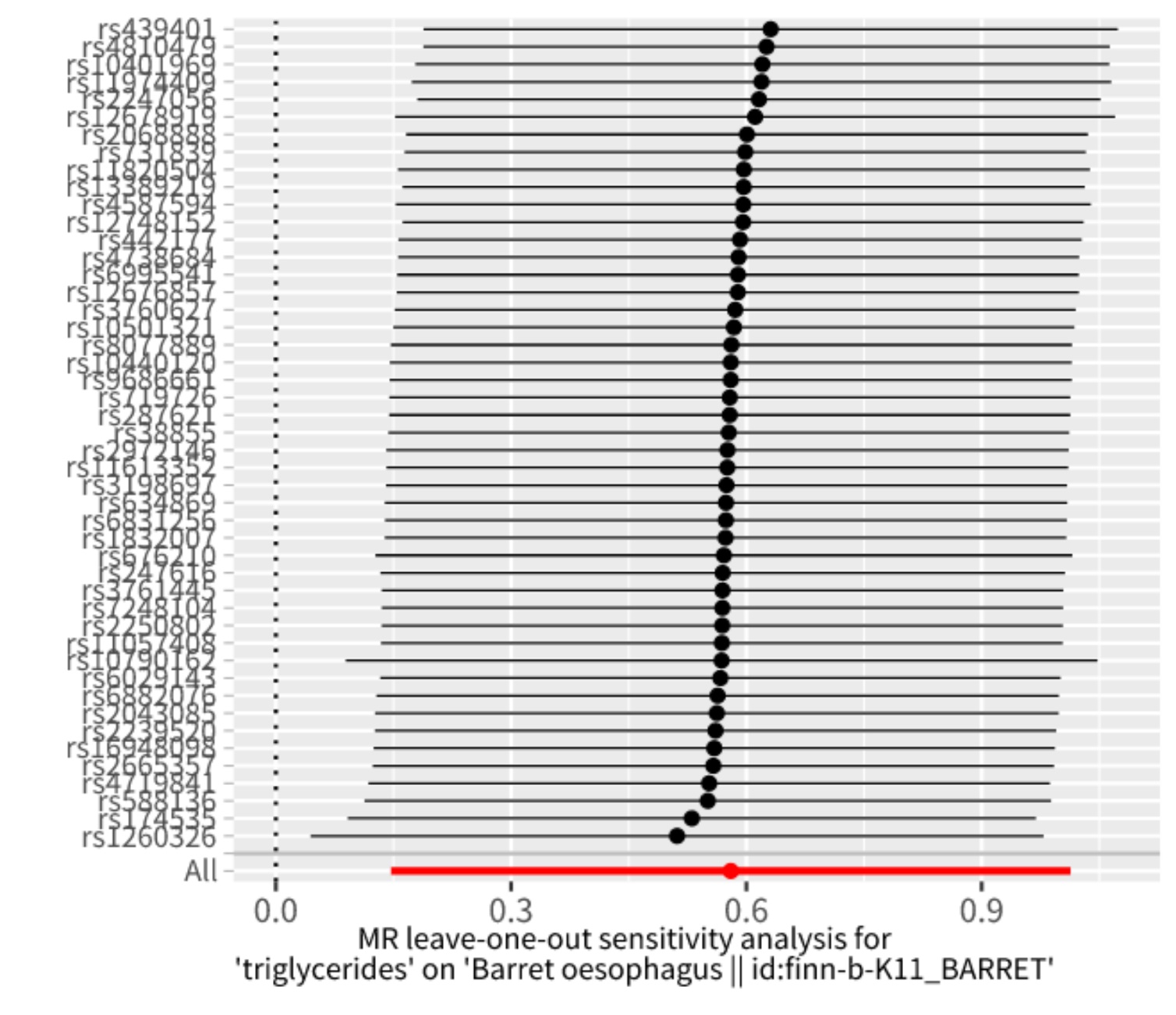


**Fig S4. The forest plot of leave-one-out sensitivity analysis for HDL cholesterol on Barrett’s Esophagus.**


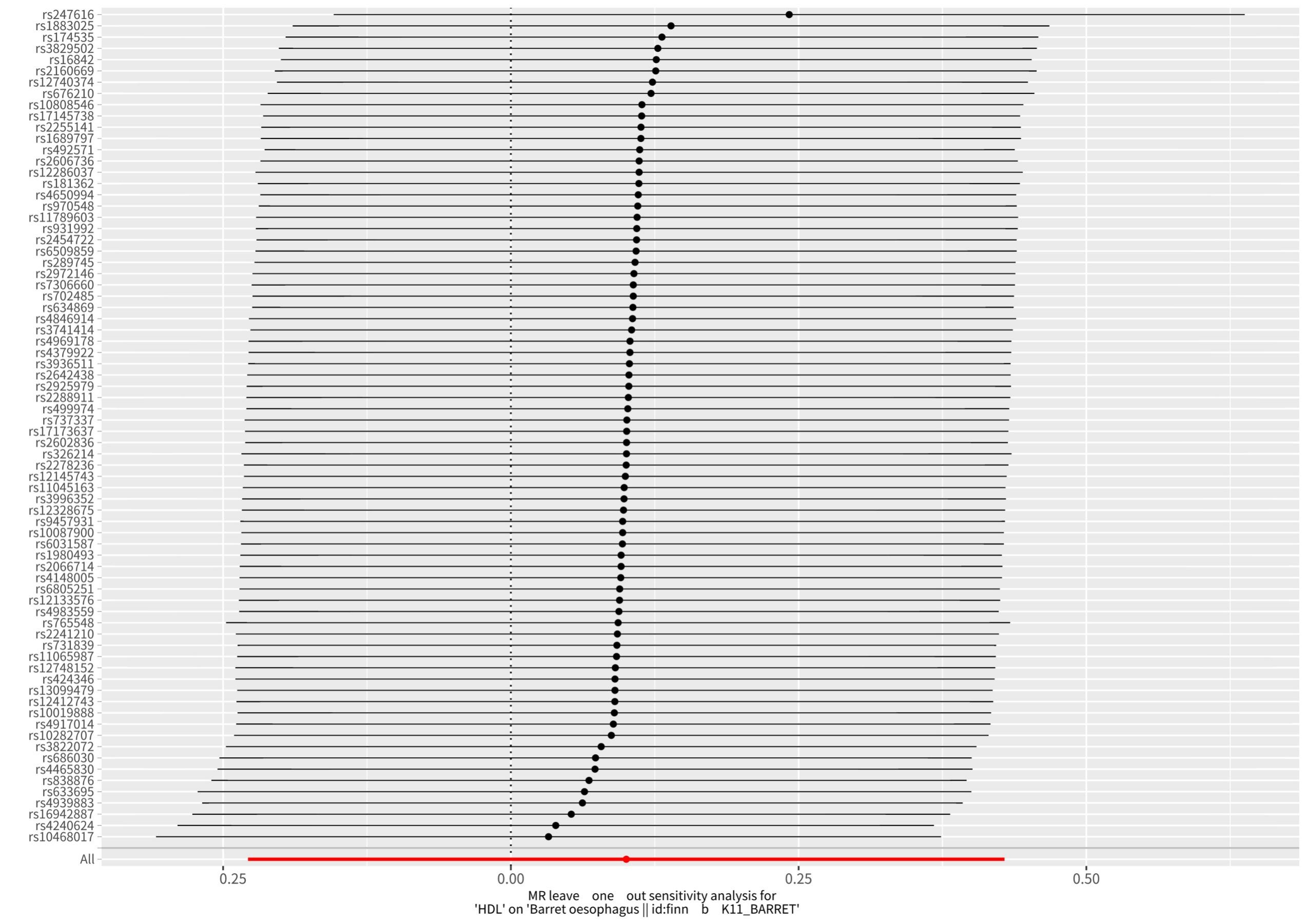


**Fig S5. The forest plot of leave-one-out sensitivity analysis for LDL cholesterol on Barrett’s Esophagus.**


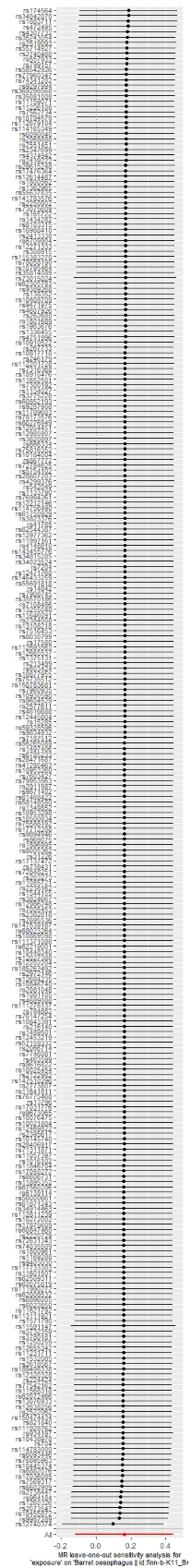


**Fig S6. The forest plot of leave-one-out sensitivity analysis for Total cholesterol on Barrett’s Esophagus.**


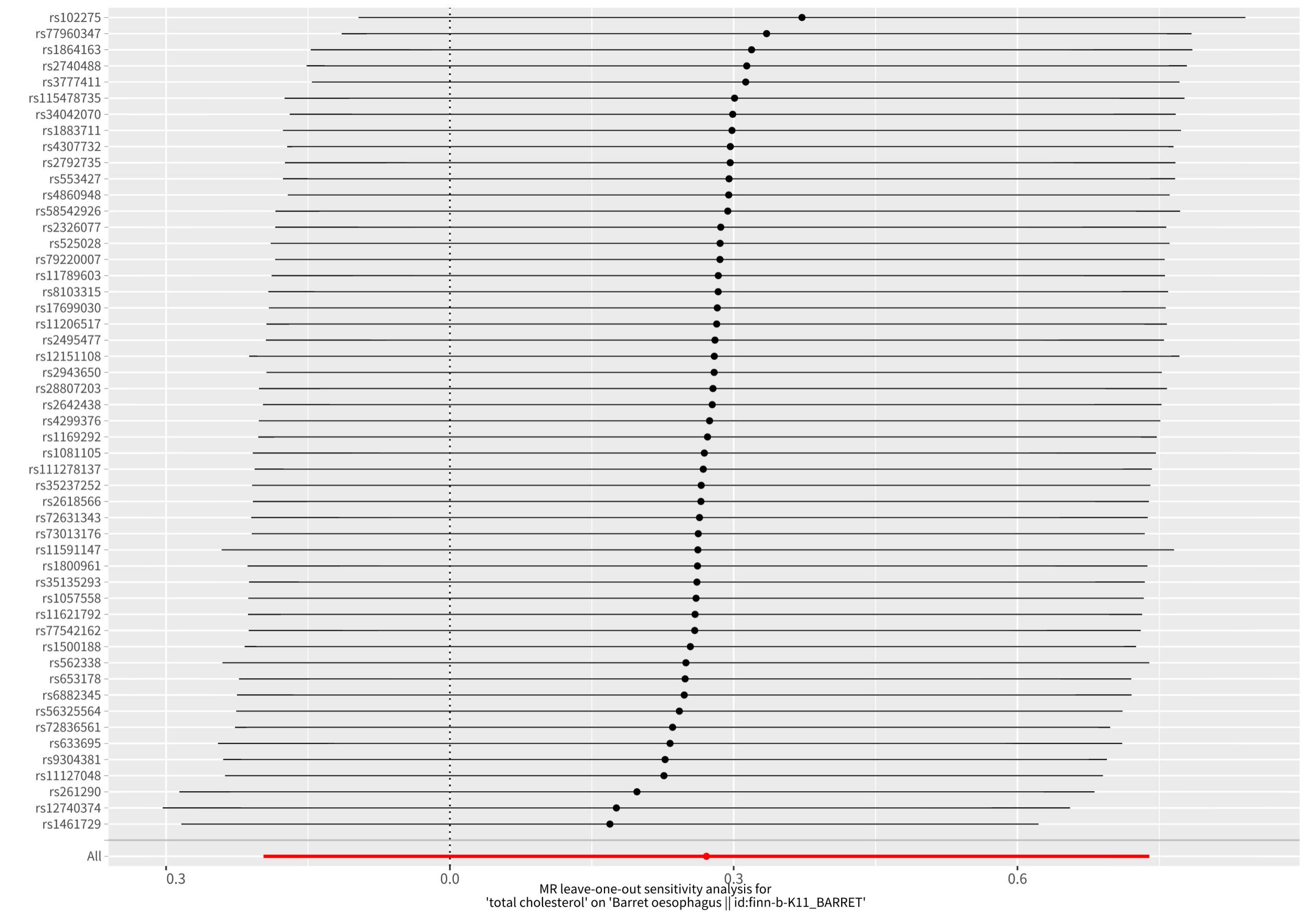


**Fig S7. The forest plot of leave-one-out sensitivity analysis for Triglycerides on Esophageal cancer.**


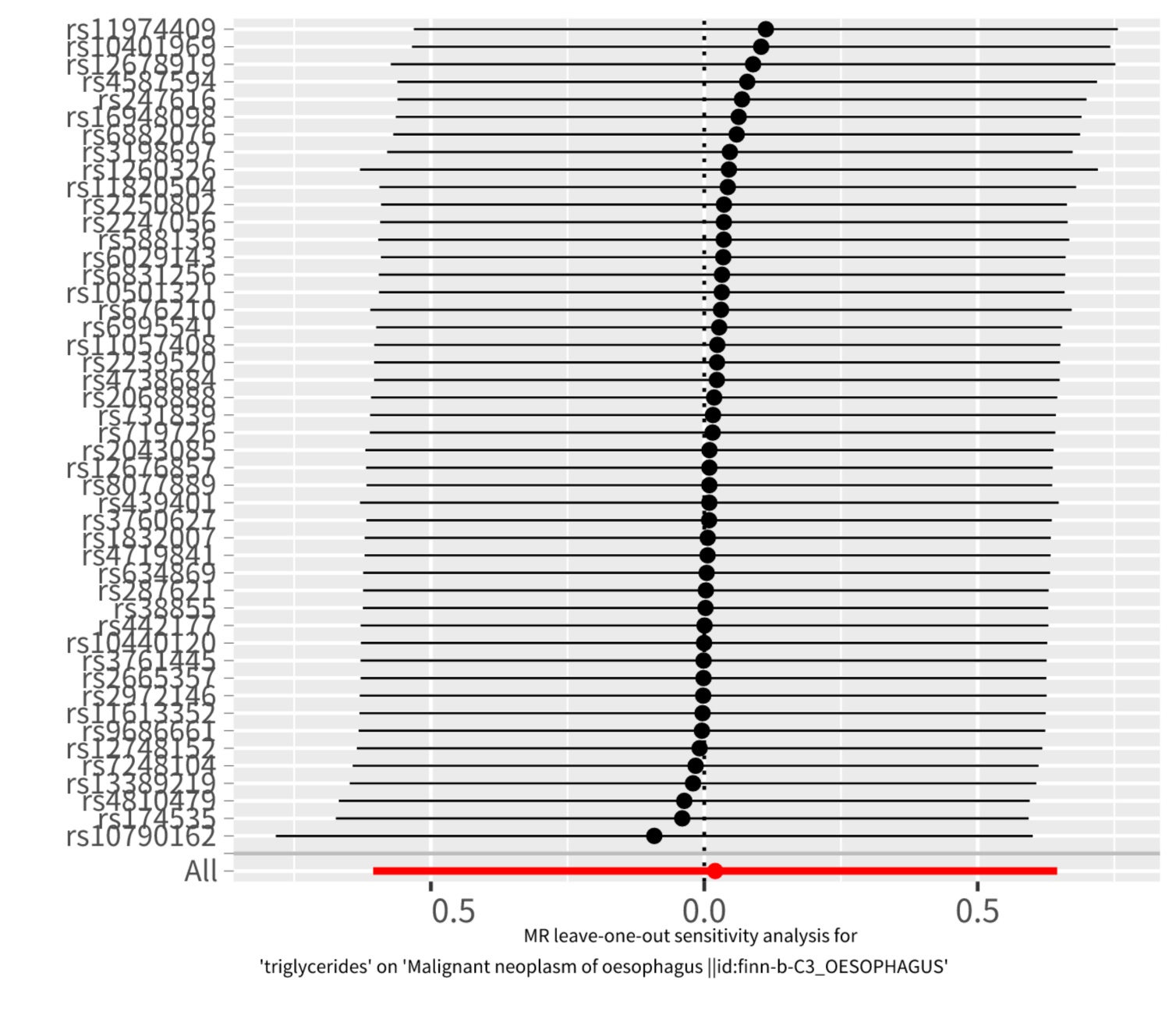


**Fig S8. The forest plot of leave-one-out sensitivity analysis for HDL cholesterol on Esophageal cancer.**


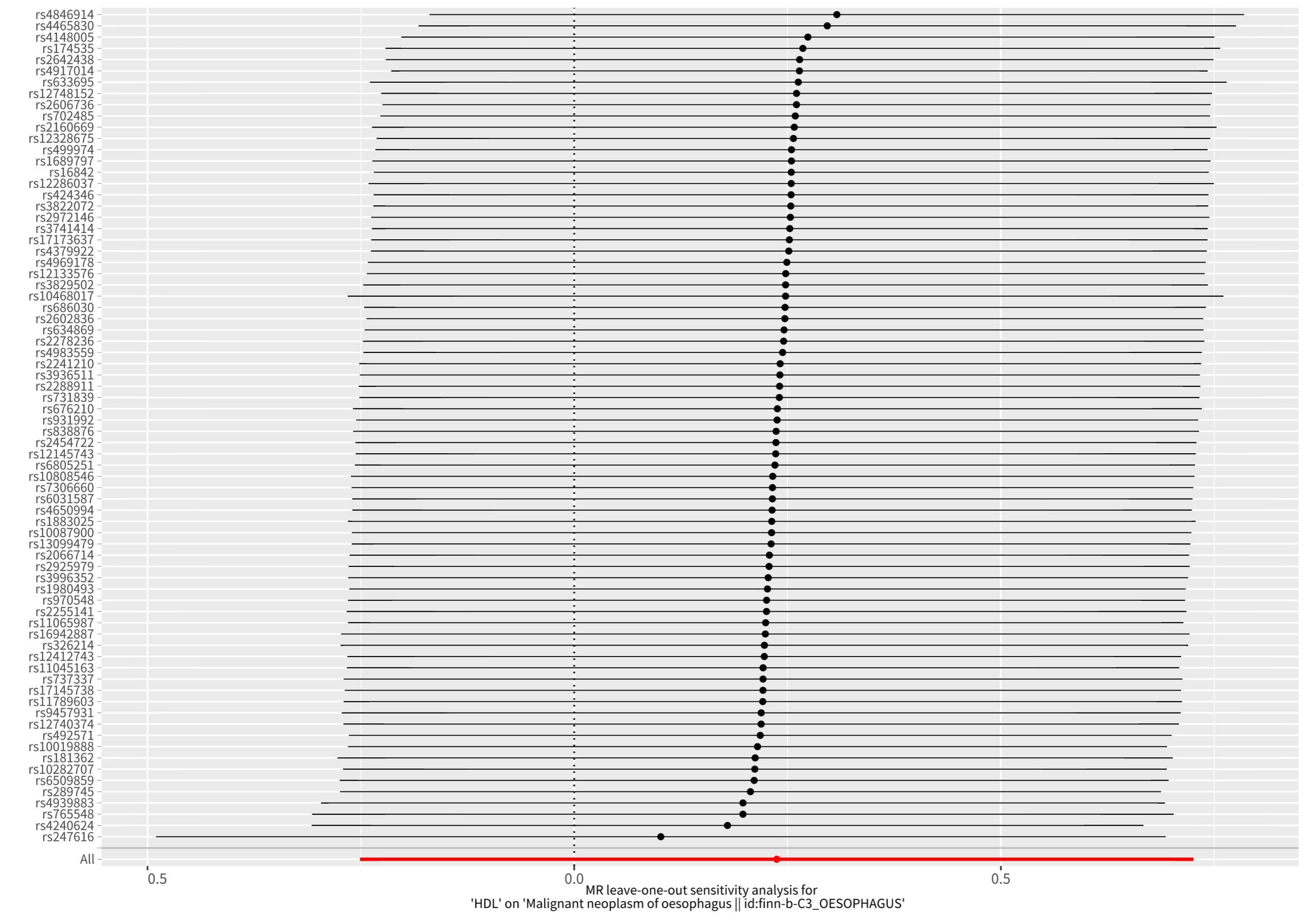


**Fig S9. The forest plot of leave-one-out sensitivity analysis for LDL cholesterol on Esophageal cancer.**


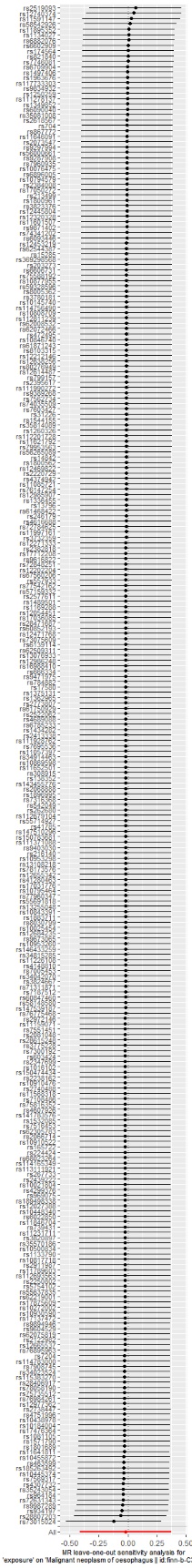


**Fig S10. The forest plot of leave-one-out sensitivity analysis for Total cholesterol on Esophageal cancer.**


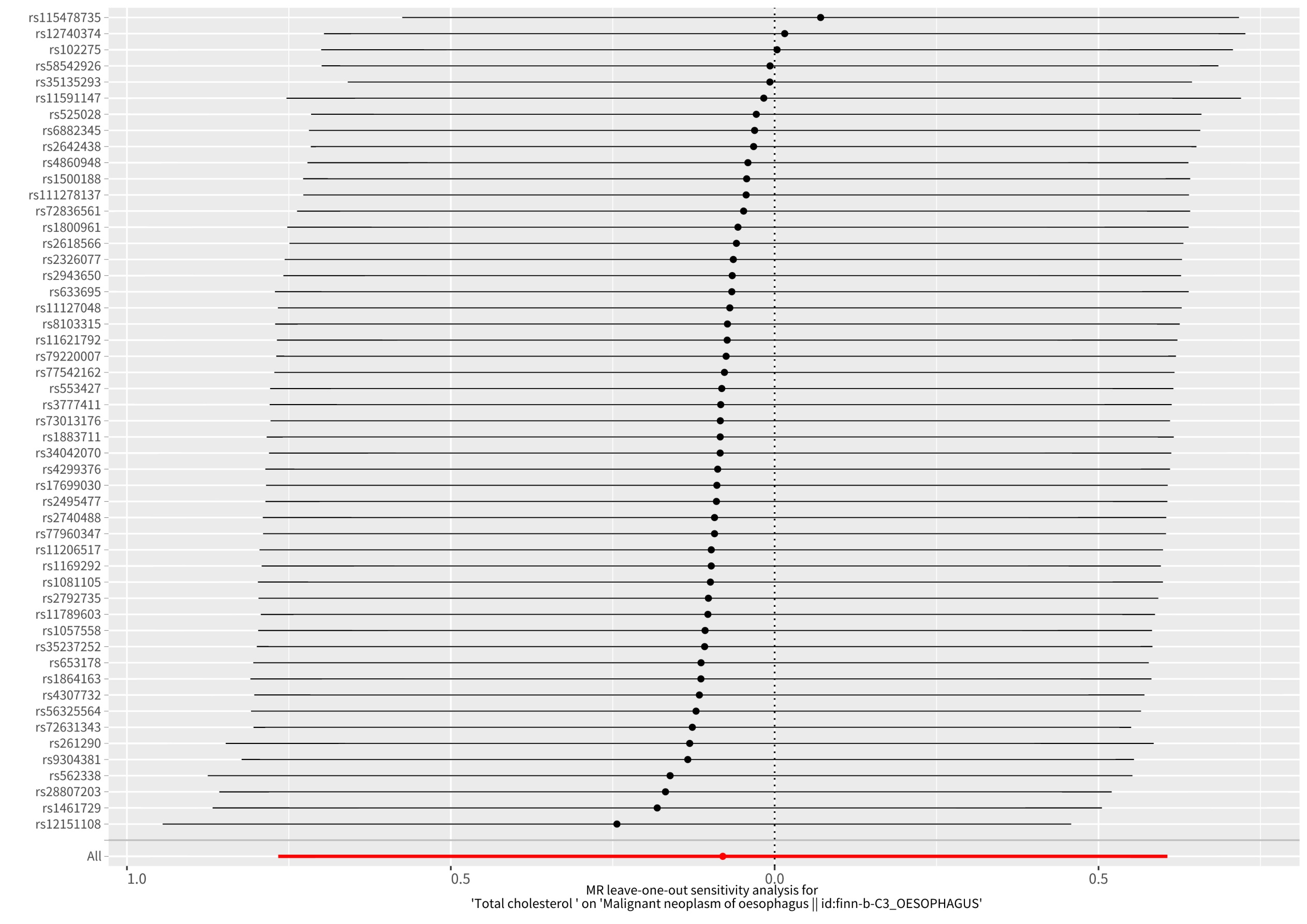

Supplement: Supplementary file 1 — Supplementary Material 1 [file 40246_2024_608_MOESM1_ESM.docx]
